# The deubiquitinase OTUD4 inhibits the expression of antimicrobial peptides in Paneth cells to support intestinal inflammation and bacterial infection

**DOI:** 10.1016/j.cellin.2023.100100

**Published:** 2023-04-05

**Authors:** Keying Yu, Yu-Yao Guo, Tianzi Liuyu, Peng Wang, Zhi-Dong Zhang, Dandan Lin, Bo Zhong

**Affiliations:** a Department of Gastrointestinal Surgery, College of Life Sciences, Zhongnan Hospital of Wuhan University, Wuhan University, Wuhan, 430071, China; b Department of Immunology, Medical Research Institute, Frontier Science Center for Immunology and Metabolism, Wuhan University, Wuhan, 430071, China; c Wuhan Research Center for Infectious Diseases and Cancer, Chinese Academy of Medical Sciences, Wuhan, 430071, China; d Cancer Center, Renmin Hospital of Wuhan University, Wuhan University, Wuhan, 430060, China; e TaiKang Center for Life and Medical Sciences, Wuhan University, Wuhan, 430071, China

**Keywords:** OTUD4, Colitis, Bacterial infection, Antimicrobial peptides, MyD88, Deubiquitination

## Abstract

Dysfunction of the intestinal epithelial barrier causes microbial invasion that would lead to inflammation in the gut. Antimicrobial peptides (AMPs) are essential components of the intestinal epithelial barrier, while the regulatory mechanisms of AMPs expression are not fully characterized. Here, we report that the ovarian tumor family deubiquitinase 4 (OTUD4) in Paneth cells restricts the expression of AMPs and thereby promotes experimental colitis and bacterial infection. OTUD4 is upregulated in the inflamed mucosa of ulcerative colitis patients and in the colon of mice treated with dextran sulfate sodium salt (DSS). Knockout of OTUD4 promotes the expression of AMPs in intestinal organoids after stimulation with lipopolysaccharide (LPS) or peptidoglycan (PGN) and in the intestinal epithelial cells (IECs) of mice after DSS treatment or *Salmonella* typhimurium (*S*.t.) infection. Consistently, *Vil*-Cre;*Otud4*^fl/fl^ mice and *Def*-Cre;*Otud4*^fl/fl^ mice exhibit hyper-resistance to DSS-induced colitis and *S*.t. infection compared to *Otud4*^fl/fl^ mice. Mechanistically, knockout of OTUD4 results in hyper K63-linked ubiquitination of MyD88 and increases the activation of NF-κB and MAPKs to promote the expression of AMPs. These findings collectively highlight an indispensable role of OTUD4 in Paneth cells to modulate AMPs production and indicate OTUD4 as a potential target for gastrointestinal inflammation and bacterial infection.

## Highlights

• Knockout of OTUD4 in IECs suppresses DSS-induced colitis and bacterial infection.
• OTUD4 restricts the expression of AMPs during intestinal inflammation and bacterial infection.
• Knockout of OTUD4 in Paneth cells suppresses DSS-induced colitis and bacterial infection by promoting AMP-production.
• OTUD4 inhibits the expression of AMPs through negatively regulating MyD88-dependent signaling.

## Graphical abstract

**Figure:**
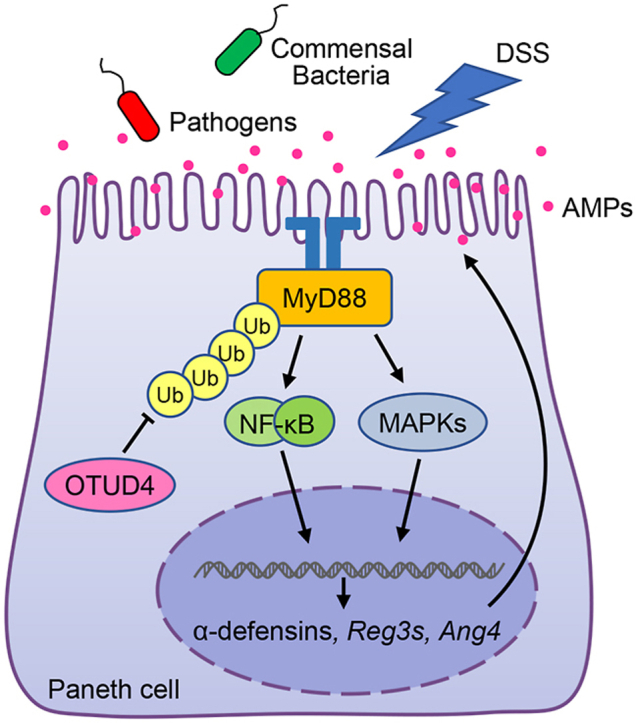

## 1 Introduction

Inflammatory bowel disease (IBD) is a chronic bowel-relapsing inflammatory disorder consisting of Crohn's disease (CD) and ulcerative colitis (UC), which has been a global health problem with a sustained increasing incidence (Ng et al., 2017). Although the specific cause of IBD remains unknown, available studies suggest that the pathogenesis of IBD is associated with genetic susceptibility, immunological abnormalities, intestinal microbiota and other environmental factors (Guan, 2019; Jeffery et al., 2022). More than two hundred genetic risk loci associated with IBD have been identified by genome-wide association studies (GWAS) and next-generation sequencing studies (Annese, 2020; Huang et al., 2017). Accumulating evidence suggests that the dysfunctions of innate and adaptive immune pathways lead to the aberrant intestinal inflammatory response in patients with IBD (Jiang et al., 2022). Infection with the pathogenic bacteria and abnormal host immune responses to the intestinal microbiota influence the development of IBD (Caruso, et al., 2020). For example, the invasive *Salmonella* triggers inflammatory responses and promotes the development of IBD (Galan, 2021; Schultz et al., 2017). Accordingly, IBD patients have a decreased intestinal microbiota abundance compared with healthy individuals. Meanwhile, the aggressive species increase and the protective species decrease in the intestinal flora of IBD patients (Caruso, et al., 2020; Forbes et al., 2018). Therefore, it is essential to maintain the homeostasis of enteric flora and inhibit the invasion of pathogenic bacteria for the prevention of IBD.

Intestinal epithelial cells (IECs) are an indispensable component of the gut which sense microbial-associated molecular patterns (MAMPs) from commensal or invading bacteria through pattern recognition receptors (PRRs) and produce antimicrobial peptides (AMPs) and cytokines/chemokines which limit pathogen propagation under homeostatic conditions or during pathological inflammation. It has been demonstrated that TLR-mediated MyD88-dependent signaling promotes the expression of AMPs and regulates bacterial infections and inflammation in the gut (Gong et al., 2010; Menendez et al., 2013). AMPs are endogenous antibiotics with antimicrobial activities, including α/β-defensins, Reg3s, lysozyme, and lectins, and are involved in intestinal inflammation and bacterial infection (Johnstone & Herzberg, 2022; Mookherjee et al., 2020). For example, mature human alpha-defensin 5 (HD-5) protects mice from *Salmonella* typhimurium (*S.*t.) and *Escherichia coli* infections and dextran sodium sulfate (DSS)-induced colitis (Ishikawa et al., 2010). AMPs are mainly produced from Paneth cells, a kind of specialized IECs with secretory functions located in the base of crypts of small intestine (Lueschow & McElroy, 2020). Paneth cells have been abnormally observed in the distal colons of IBD patients and their dysfunction in AMP and lysosome production contributes to the pathogenesis of experimental colitis in mice (Yu et al., 2020; Zhang & Liu, 2016). Therefore, it is of great interest to identify the key regulatory proteins in modulating the production of AMPs in Paneth cells.

Deubiquitinases (DUBs) counteract ubiquitination by cleaving poly- or mono-ubiquitin from target proteins and play critical roles in various biological processes. To date, six families of DUBs have been reported, including the ubiquitin-specific proteases (USPs), the ovarian tumor proteases (OTUs), the ubiquitin C-terminal hydrolases (UCHs), the Josephin family, the JAB1/MPN/MOV34 metalloproteases (JAMMs), and the motif interacting with Ub-containing novel DUB family (MINDYs) (Mevissen & Komander, 2017). Ovarian tumor family deubiquitinase 4 (OTUD4) belongs to the OTU family and is involved in regulating embryogenesis of zebrafish (Tse et al., 2013), DNA alkylation damage repair (Zhao et al., 2015), RNA-binding and translation (Das et al., 2019), neural progenitor self-renewal and neurogenesis (Kedia et al., 2022). In addition, we and others have found that virus-triggered induction of OTUD4 removes K48-linked polyubiquitin chains on MAVS to promote antiviral responses and that phosphorylated OTUD4 removes K63-linked polyubiquitin chains on MyD88 resulting in downregulation of TLR-mediated NF-κB signaling (Liuyu et al., 2019; Zhao, et al., 2018). However, whether and how OTUD4 regulates intestinal inflammation and antibacterial response remains unexplored.

Here we report that OTUD4 deficiency in IECs or Paneth cells leads to resistance to DSS-induced colitis and *S*.t. infection. We further show that OTUD4 negatively regulates the expression of AMPs in IECs after DSS treatment or bacterial infection in a MyD88-dependent manner. These findings indicate that OTUD4 plays an essential role in intestinal inflammation and bacterial infection and serves as a potential therapeutic target for the related diseases.

## 2 Results

### 2.1 Knockout of OTUD4 in IECs alleviates DSS-induced colitis

**Fig. 1:**
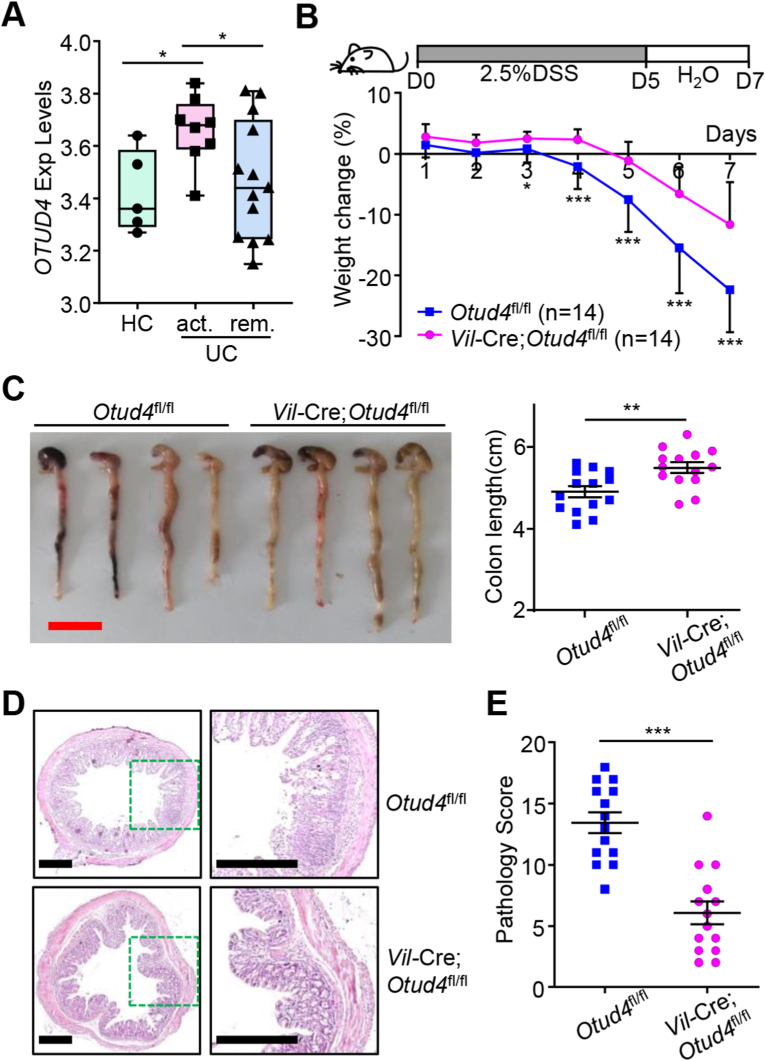
**Otud4 deficiency in IECs leads to increased resistance toDSS-inducedcolitis.** (A) *OTUD4* mRNA expression levels in colonic mucosa tissues from ulcerative colitis (UC) patients and healthy individuals (data from GEO database GDS3119) (n = 26). (B) A scheme of DSS-induced colitis (upper scheme) and body weight change (lower graph) of *Otud4*^fl/fl^ (n = 14) and *Vil*-Cre;*Otud4*^fl/fl^ (n = 14) mice treated with 2.5% DSS for 5 days followed by normal sterile water for 2 days. (C) A representative image and the lengths of colons of *Otud4*^fl/fl^ (n = 14) and *Vil*-Cre;*Otud4*^fl/fl^ (n = 14) mice treated as in (B). (D) Representative images of HE-stained colon sections of *Otud4*^fl/fl^ and *Vil*-Cre;*Otud4*^fl/fl^ mice treated as in (B). (E) The pathology scores of *Otud4*^fl/fl^ (n = 14) and *Vil*-Cre;*Otud4*^fl/fl^ (n = 14) mice treated as in (B). ∗*P* ​< ​0.05, ∗∗*P* ​< ​0.01, ∗∗∗*P* < 0.001 (Student's unpaired *t*-test in A–C, E). Graphs show mean ± S.D. (A–C, E). Red and black scale bars represent 2 cm and 400 μm, respectively (C, D). Data are combined three independent experiments (B, C, E) or representative of three independent experiments (D).

To explore whether OTUD4 plays a role in intestinal inflammation, we first analyzed the transcriptomic data of the colon tissues from patients with UC. The results showed that *OTUD4* was highly expressed in the inflammatory mucosa of patients with active UC compared to the mucosa from healthy people or remissive UC patients (Fig. 1A). Further analysis of single-cell transcriptomic data from UC patients suggested that OTUD4 was expressed in epithelial cells and monocytes in the ulcerative mucosa of UC patients (Smillie et al., 2019) (Fig. S1A). We next examined whether myeloid depletion of OTUD4 affected DSS-induced colitis in mice. The *Lyz2*-Cre;*Otud4*^fl/fl^ and the *Otud4*^fl/fl^ mice were fed with 2.5% DSS for 5 successive days and then with normal sterile water for 2 days and the body weight and the shape and blood in stool were observed and recorded daily during this period (An et al., 2022; Liuyu et al., 2019; Wang et al., 2020; Zhao, Wang, et al., 2018). The *Lyz2*-Cre;*Otud4*^fl/fl^ mice exhibited similar weight loss compared to *Otud4*^fl/fl^ mice during colitis induction (Fig. S1B). In addition, the colon shortening was comparable between the *Lyz2*-Cre;*Otud4*^fl/fl^ and the *Otud4*^fl/fl^ mice after DSS treatment (Fig. S1C). Results from hematoxylin and eosin (H&E) staining of the inflamed colons showed that the *Lyz2*-Cre;*Otud4*^fl/fl^ and the *Otud4*^fl/fl^ mice exhibited similar levels of epithelial damage and leukocyte infiltration in the colon (Fig. S1D). Pathogenic scores of colitis which were evaluated by combining disease activity index (DAI) score and histopathological score also suggested similar pathology of the *Lyz2*-Cre;*Otud4*^fl/fl^ and the *Otud4*^fl/fl^ mice after DSS treatment (Fig. S1E), indicating that OTUD4 in myeloid cells plays a dispensable role in DSS-induced colitis.

Considering that IECs play an important role in intestinal inflammation and OTUD4 is highly expressed in IECs after DSS treatment (Fig. S2A), we crossed the *Otud4*^fl/fl^ mice with the *Vil*-Cre mice to obtain the *Vil*-Cre;*Otud4*^fl/fl^ mice in which OTUD4 was specifically deleted in IECs (An et al., 2022). Results from qRT-PCR, immunoblot, and immunohistochemistry (IHC) assays demonstrated that OTUD4 was significantly downregulated in IECs of the *Vil*-Cre;*Otud4*^fl/fl^ mice (Figs. S2B–D), indicating an efficient and specific depletion of OTUD4 in the IECs of *Vil*-Cre;*Otud4*^fl/fl^ mice. We further found that the numbers of Paneth cells (lysozyme^+^) in the crypts were significantly increased in the small intestines of the *Vil*-Cre;*Otud4*^fl/fl^ mice compared to *Otud4*^fl/fl^ mice, while the number of goblet cells (AB/PAS^+^) and the proliferative cells (Ki67^+^) in small intestines and colons were comparable between the *Vil*-Cre;*Otud4*^fl/fl^ and the *Otud4*^fl/fl^ mice before or after DSS treatment (Figs. S3A and S3B). We next induced colitis in these mice and observed that the *Vil*-Cre;*Otud4*^fl/fl^ mice exhibited increased resistance to weight loss, diarrhea, and colon shortening compared to *Otud4*^fl/fl^ mice (Fig. 1B and C). Results from H&E staining of the colons and pathogenic scoring suggested that the *Vil*-Cre;*Otud4*^fl/fl^ mice had less leukocyte infiltration, more intact epithelium, and less severe pathologic symptoms than *Otud4*^fl/fl^ mice after DSS treatment (Fig. 1D and E). Collectively, these data suggest that the knockout of OTUD4 in IECs inhibits colon inflammation after DSS treatment.

### 2.2 OTUD4 in IECs is dispensable for colon cancerogenesis

Chronic inflammation including long-standing UC and Crohn's colitis is a major driver of neoplastic progression, resulting in dysplastic precursor lesions and increased risk of developing colorectal cancer (CRC) (Shah & Itzkowitz, 2022). We next investigated the role of OTUD4 in colon cancer development. In the azoxymethane (AOM)/DSS model of colon cancer, the *Vil*-Cre;*Otud4*^fl/fl^ and the *Otud4*^fl/fl^ mice exhibited comparable tumor numbers and roughly equal mortality rates (Figs. S4A and S4B). In the AOM/*Vil*-Cre;*Trp53*^fl/fl^ (VP) model, the deficiency of OTUD4 in IECs had little effect on tumor numbers or mortality rates (Figs. S4C and S4D). These results imply that the loss of OTUD4 in IECs does not inhibit the progression of colon cancer in the AOM/DSS and AOM/VP models.

### 2.3 OTUD4 deficiency in IECs promotes the expression of AMPs and alters the microbiota after DSS treatment

**Fig. 2:**
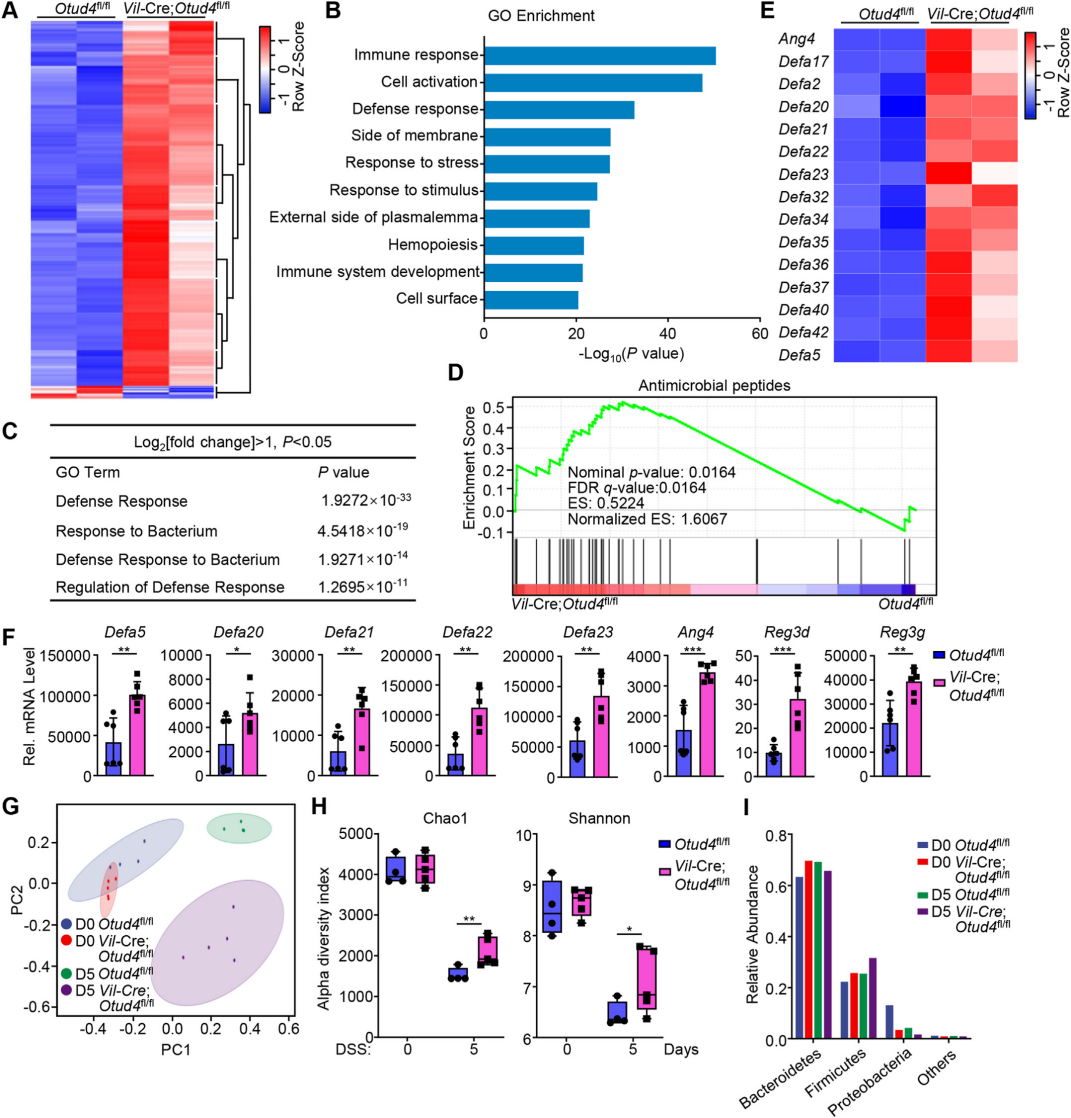
**Depletion of OTUD4 in IECs alters the expression of AMPs and reshapes gut microbiota.** (A) Heat map of differentially expressed genes (DEGs) (*P* < 0.05 and |log_2_ Fold change|≥1) in the transcriptomic profile of intestinal epithelial cells (IECs) from *Otud4*^fl/fl^ (n = 2) and *Vil*-Cre;*Otud4*^fl/fl^ (n = 2) mice that were given 2.5% DSS in drinking water for 2 d. (B) GO (gene ontology) analysis from the transcriptomic data obtained in (A). (C) A table of GO pathways associated with bacterial defense from the transcriptomic data obtained in (A). (D) GSEA analysis of antimicrobial peptides from the transcriptomic data obtained in (A). FDR, false discovery rate; ES, enrichment score. (E) Heatmap of the indicated genes related antimicrobial peptides from the transcriptomic data obtained in (A). (F) qRT-PCR analysis of the indicated genes of IECs from two groups of *Otud4*^fl/fl^ (n = 6) and *Vil*-Cre;*Otud4*^fl/fl^ (n = 6) mice that were given 2.5% DSS in drinking water for 2 d. (G) PCoA analysis from 16S rRNA sequencing in the feces of *Otud4*^fl/fl^ (n = 4) and *Vil*-Cre;*Otud4*^fl/fl^ (n = 5) mice that were given 2.5% DSS in drinking water for 0 d or 5 d. (H) Chao1 richness and Shannon diversity analysis of the 16S rRNA sequencing data obtained in (G). (I) Bacterial relative abundance from 16S rRNA sequencing data obtained in (G). ∗*P* ​< ​0.05, ∗∗*P* ​< ​0.01, ∗∗∗*P* < 0.001 (Student's unpaired *t*-test in F, H). Graphs show mean ± S.D. (F, H). Data are representative of two (F) independent experiments.

To investigate the mechanism by which the epithelial OTUD4 regulates DSS-induced colitis, we isolated the IECs from *Vil*-Cre;*Otud4*^fl/fl^ and *Otud4*^fl/fl^ mice that were given 2.5% DSS in drinking water for 2 days and performed transcriptomic sequencing assays. Results from transcriptomic analysis showed that 623 genes (602 upregulated and 21 downregulated) were differentially expressed with statistical significance in IECs from *Vil*-Cre;*Otud4*^fl/fl^ mice compared to *Otud4*^fl/fl^ mice (Fig. 2A). Gene ontology (GO) analysis identified several pathways (threshold *P* value < 0.05) such as pathways associated with defense response and response to bacterium significantly enriched in the upregulated gene sets (Fig. 2B and C). Gene-set enrichment analysis (GSEA) characterized the differentially expressed genes into clusters of AMPs with antibacterial activities (Fig. 2D and E). Results from qRT-PCR analysis further confirmed that knockout of OTUD4 in IECs led to increased expression of AMPs after DSS treatment (Fig. 2F). These data suggest that OTUD4 in IECs negatively regulates the expression of AMPs after DSS treatment.

Because dysbiosis and alterations in the intestinal microbiota are associated with the progress of IBD and AMPs play an important role in restricting the invasion of the microbes in gut (Muniz et al., 2012), we collected feces from the *Vil*-Cre;*Otud4*^fl/fl^ and the *Otud4*^fl/fl^ mice which were given 2.5% DSS in drinking water for 0 or 5 days, and analyzed the microbial composition in the feces by 16S rRNA sequencing assays. The results showed that the composition of microbiota in feces of the *Vil*-Cre;*Otud4*^fl/fl^ and the *Otud4*^fl/fl^ mice were significantly differed after DSS treatment (Fig. 2G). The richness and diversity of the microbiota in feces of the *Vil*-Cre;*Otud4*^fl/fl^ mice were less decreased than in those of the *Otud4*^fl/fl^ mice after DSS treatment (Fig. 2H). Further analysis revealed that the *Vil*-Cre;*Otud4*^fl/fl^ mice shaped more microbes of the phylum Firmicutes and fewer microbes of the phylum Proteobacteria than the *Otud4*^fl/fl^ mice after DSS treatment (Fig. 2I). It has been demonstrated that phylum Firmicutes metabolize polysaccharides into short-chain fatty acids as the primary carbon energy source for colonocytes and are beneficial for host, while an increased prevalence of Proteobacteria is a potential diagnostic signature of dysbiosis and risk of disease (La Rosa et al., 2019; Shin et al., 2015; Simpson & Campbell, 2015), indicating that a healthier composition of gut bacteria in the *Vil*-Cre;*Otud4*^fl/fl^ mice than the *Otud4*^fl/fl^ mice during DSS-induced colitis. Together, these results indicate that OTUD4 deficiency in IECs results in resistance to dysregulation of microbiota in the gut after DSS treatment.

### 2.4 Depletion of OTUD4 in IECs inhibits bacterial infection in the gut

**Fig. 3:**
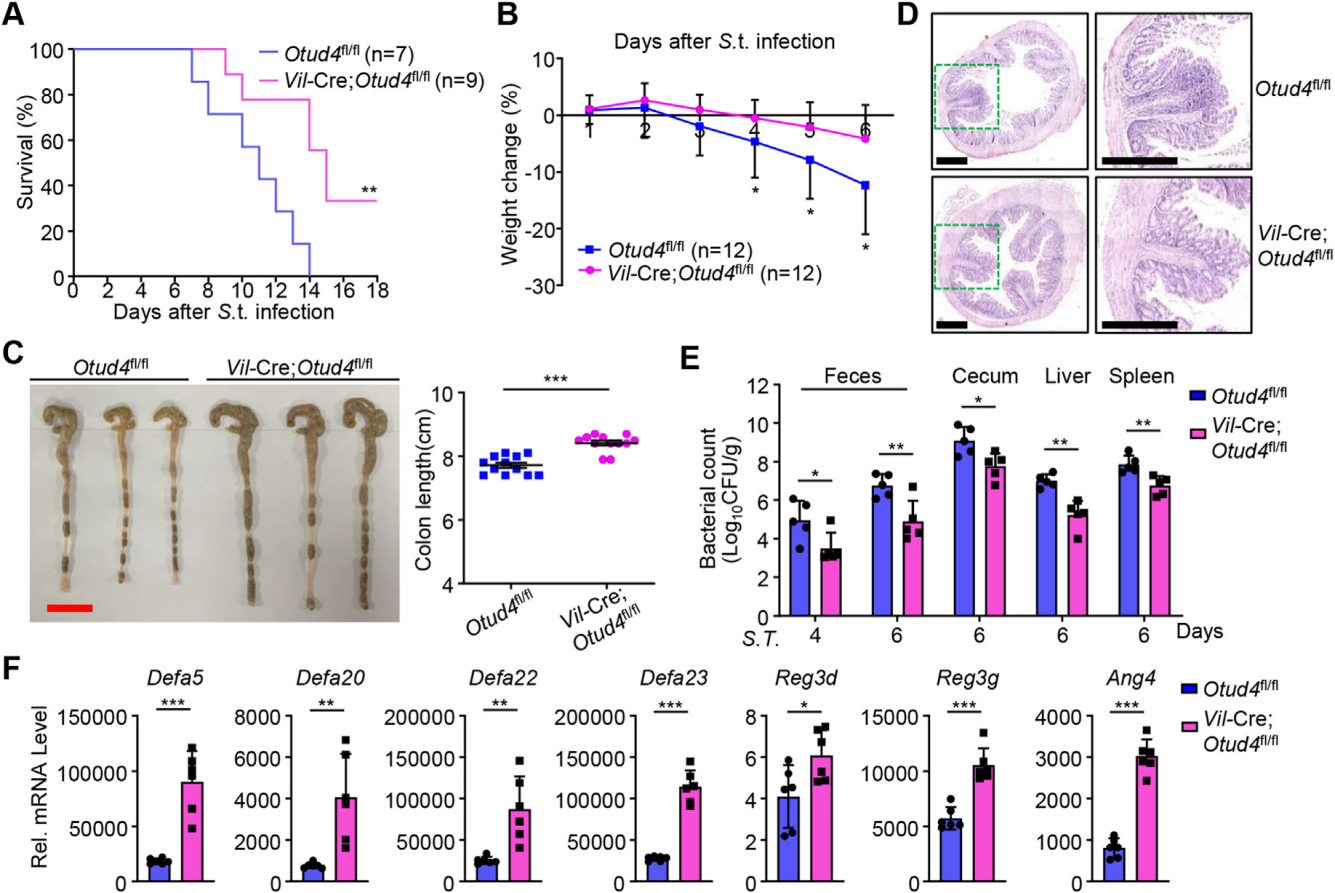
**Knockout of OTUD4 in IECs leads tohyper-resistanceto*****S*****.t. infection.** (A) Survival of *Otud4*^fl/fl^ (n = 7) and *Vil*-Cre;*Otud4*^fl/fl^ (n = 9) mice that were injected with *Salmonella* typhimurium (*S*.t.) (2 × 10^7^ c.f.u. per mouse) by gavage. (B) Body weight change of *Otud4*^fl/fl^ (n = 12) and *Vil*-Cre;*Otud4*^fl/fl^ (n = 12) mice treated as in (A). (C) A representative image and the lengths of colons of *Otud4*^fl/fl^ (n = 12) and *Vil*-Cre;*Otud4*^fl/fl^ (n = 12) mice treated as in (A) and sacrificed at day 6. (D) Representative images of HE-stained colon sections of *Otud4*^fl/fl^ and *Vil*-Cre;*Otud4*^fl/fl^ mice treated as in (A) and sacrificed at day 6. (E) Bacterial counts (CFU/g) of feces, cecum, liver, and spleen from *Otud4*^fl/fl^ (n = 5) and *Vil*-Cre;*Otud4*^fl/fl^ (n = 5) mice treated as in (A). (F) qRT-PCR analysis of expression levels of the indicated genes of IECs from *Otud4*^fl/fl^ (n = 6) and *Vil*-Cre;*Otud4*^fl/fl^ (n = 6) mice at day 2 after injection of *S*.t. (2 × 10^7^ c.f.u. per mouse) by gavage. ∗*P* ​< ​0.05, ∗∗*P* ​< ​0.01, ∗∗∗*P* < 0.001 (Log-rank test in A, Student's unpaired *t*-test in B, C, E, F). Graphs show mean ± S.D. (B, C, E, F). Red and black scale bars represent 2 cm and 400 μm, respectively (C, D). Data are representative of two independent experiments (A, D–F) or combined two independent experiments (B, C).

DSS treatment causes intestinal epithelium damage that leads to the invasion of commensal bacteria and severe inflammation (Strober et al., 2007), and some enteric pathogens such as *Salmonella* Typhimurium (S.t.) trigger intestinal inflammation to sustain its replication in the intestinal tract (Galan, 2021). Because OTUD4 negatively regulates the expression of AMPs during DSS-induced colitis, we next investigated whether OTUD4 regulated antibacterial responses in the gut. The *Vil*-Cre;*Otud4*^fl/fl^ and the *Otud4*^fl/fl^ mice were infected with *S*.t. by gavage and the survival and body weight were monitored. We found that the *Vil*-Cre;*Otud4*^fl/fl^ mice had higher survival rates, less weight loss, and longer colons in comparison to *Otud4*^fl/fl^ mice (Fig. 3A–C). Results from H&E staining showed that colons from *Vil*-Cre;*Otud4*^fl/fl^ mice exhibited decreased inflammation compared to those from the *Otud4*^fl/fl^ mice (Fig. 3D). The bacterial counts in feces, cecum, liver and spleen of the *Vil*-Cre;*Otud4*^fl/fl^ mice were also significantly reduced compared to *Otud4*^fl/fl^ mice (Fig. 3E), indicating that OTUD4 promotes bacterial infection *in vivo*. Consistent with these observations, OTUD4 deficiency in IECs promoted the expression of AMPs after *S*.t. infection (Fig. 3F). Taken together, our results indicate that OTUD4 in IECs promotes bacterial infection by inhibiting the expression of AMPs.

### 2.5 OTUD4 in Paneth cells promotes intestinal inflammation and bacterial infection

Paneth cells are specialized AMP-producing cells in the epithelium of the small intestine and dysregulation of AMP production in Paneth cells contributes to the pathogenesis of IBD (Adolph et al., 2013; Clevers & Bevins, 2013; Ghosh et al., 2002). Consistent with the observations that more Paneth cells existed in the crypts of small intestines of *Vil*-Cre;*Otud4*^fl/fl^ mice than in those of *Otud4*^fl/fl^ mice (Figs. S3A and S3B), the basal expression levels of AMPs such as *Defa5*, *Defa22*, *Reg3d* and *Reg3g* were significantly higher in *Vil*-Cre;*Otud4*^fl/fl^ IECs than in *Otud4*^fl/fl^ IECs (Fig. S5A). To more specifically define the intrinsic role of OTUD4 in the regulation of AMPs and intestinal inflammation, we crossed the *Otud4*^fl/fl^ mice with the defensinα6-Cre (*Def*-Cre) mice to obtain the *Def*-Cre;*Otud4*^fl/fl^ mice in which OTUD4 was specifically deleted in Paneth cells (Wang et al., 2021). Interestingly, the numbers of Paneth cells in the crypts of small intestines of *Def*-Cre;*Otud4*^fl/fl^ mice were comparable to those of *Otud4*^fl/fl^ mice (Figs. S5B and S5C), indicating that depletion of OTUD4 in Paneth cells does not affect the differentiation or maintenance of Paneth cells.

**Fig. 4:**
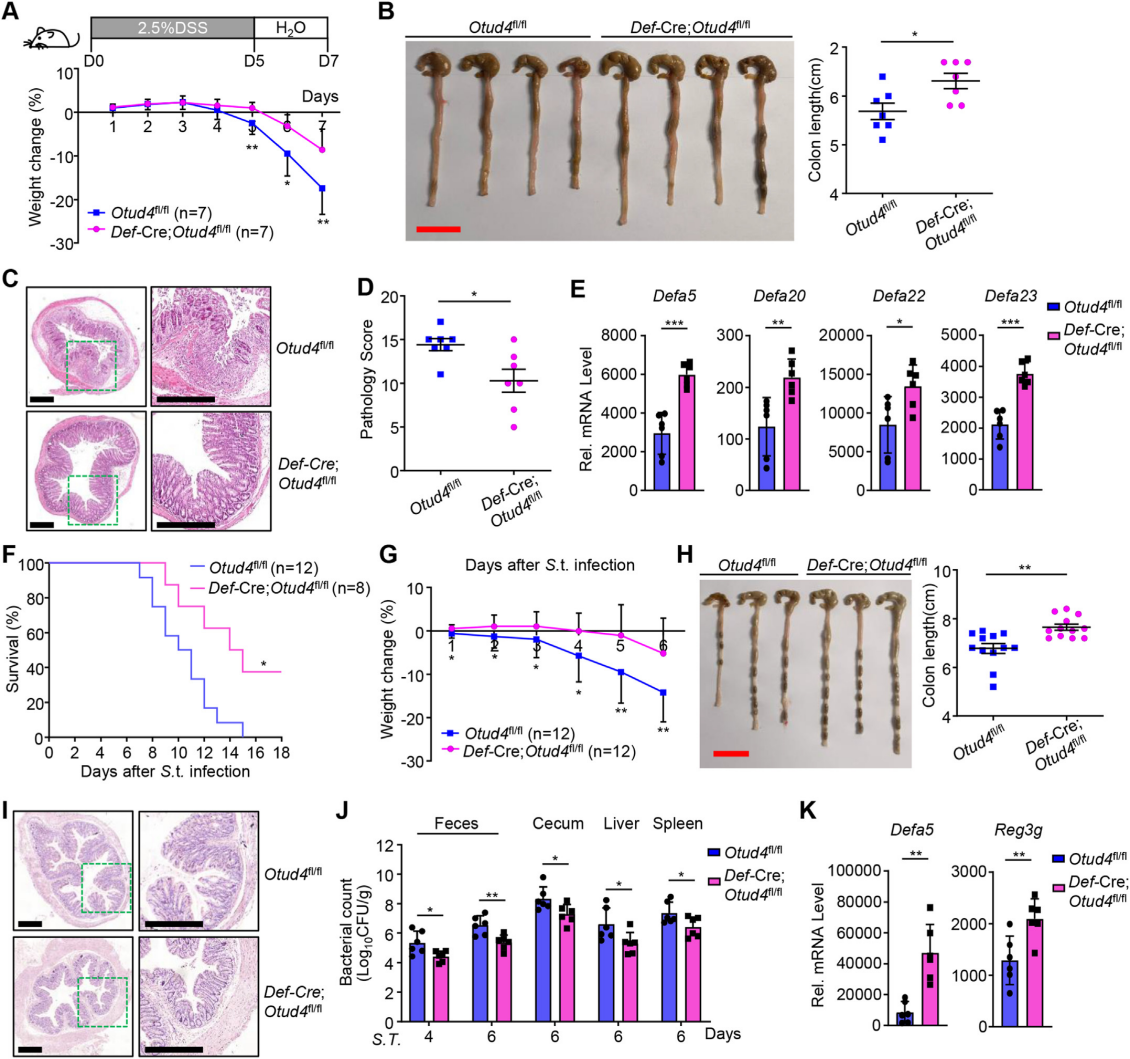
**OTUD4 in Paneth cells supportsDSS-induced colitis and*****S*****.t. infection.** (A) A scheme of DSS-induced colitis (upper scheme) and body weight change (lower graph) of *Otud4*^fl/fl^ (n = 7) and *Def*-Cre;*Otud4*^fl/fl^ (n = 7) mice with 2.5% DSS for 5 days followed by normal sterile water for 2 days. (B) A representative image and the lengths of colons of *Otud4*^fl/fl^ (n = 7) and *Def*-Cre;*Otud4*^fl/fl^ (n = 7) mice treated as in (A). (C) Representative images of HE-stained colon sections of *Otud4*^fl/fl^ and *Def-*Cre;*Otud4*^fl/fl^ mice treated as in (A). (D) The pathology scores of *Otud4*^fl/fl^ (n = 7) and *Def*-Cre;*Otud4*^fl/fl^ (n = 7) mice treated as in (A). (E) qRT-PCR analysis of expression levels of the indicated genes of IECs from *Otud4*^fl/fl^ (n = 6) and *Def*-Cre;*Otud4*^fl/fl^ (n = 6) mice that were given 2.5% DSS in drinking water for 2 d. (F) Survival of *Otud4*^fl/fl^ (n = 12) and *Def*-Cre;*Otud4*^fl/fl^ (n = 8) mice that were injected with *S*.t. (2 × 10^7^ c.f.u. per mouse) by gavage. (G) Body weight change of *Otud4*^fl/fl^ (n = 12) and *Def*-Cre;*Otud4*^fl/fl^ (n = 12) mice treated as in (F) and sacrificed at day 6. (H) A representative image and the lengths of colons of *Otud4*^fl/fl^ (n = 12) and *Def*-Cre;*Otud4*^fl/fl^ (n = 12) mice treated as in (F) and sacrificed at day 6. (I) Representative images of HE-stained colon sections of *Otud4*^fl/fl^ and *Def*-Cre;*Otud4*^fl/fl^ mice treated as in (F) and sacrificed at day 6. (J) Bacterial counts (CFU/g) of feces, cecum, liver, and spleen from *Otud4*^fl/fl^ (n = 6) and *Def*-Cre;*Otud4*^fl/fl^ (n = 6) mice treated as in (F). (K) qRT-PCR analysis of *Def5a* or *Reg3g* of IECs from *Otud4*^fl/fl^ (n = 6) and *Def*-Cre;*Otud4*^fl/fl^ (n = 6) mice at day 2 after injection of *S*.t. (2 × 10^7^ c.f.u. per mouse) by gavage. ∗*P* ​< ​0.05, ∗∗*P* ​< ​0.01, ∗∗∗*P* < 0.001 (Student's unpaired *t*-test in A, B, D, E, G, H, J, K, Log-rank test in F). Graphs show mean ± S.D. (A, B, D, E, G, H, J, K). Red and black scale bars represent 2 cm and 400 μm (B, C, H, I). Data are combined two independent experiments (A, B, D, F–H) or representative of two independent experiments (C, E, I, J, K).

In the DSS-induced colitis model, the *Def*-Cre;*Otud4*^fl/fl^ mice exhibited increased resistance to weight loss, diarrhea, and colon shortening compared to *Otud4*^fl/fl^ mice (Fig. 4A and B). Results from H&E staining of the colons and pathogenic scores showed that the *Def*-Cre;*Otud4*^fl/fl^ mice had less leukocyte infiltration, more intact epithelium, and less severe pathologic symptoms than *Otud4*^fl/fl^ mice after DSS treatment (Fig. 4C and D). Results from qRT-PCR assays suggested that OTUD4 deficiency in Paneth cells promoted the expression of AMPs in the intestinal epithelium after DSS treatment (Fig. 4E), suggesting that OTUD4 in Paneth cells promotes intestinal inflammation.

We next infected *Def*-Cre;*Otud4*^fl/fl^ and *Otud4*^fl/fl^ mice with *S*.t. and found that the *Def*-Cre;*Otud4*^fl/fl^ mice exhibited increased survival and decreased weight loss compared to *Otud4*^fl/fl^ mice after lethal *S*.t. infection (Fig. 4F and G), indicating that loss of OTUD4 in Paneth cells leads to hyper-resistance to *S*.t. infection. In addition, the *Def*-Cre;*Otud4*^fl/fl^ mice exhibited less colon shortening and more integral crypts in the colon than the *Otud4*^fl/fl^ mice after *S*.t. infection (Fig. 4. H and I), indicating less inflammation in the gut of *Def*-Cre;*Otud4*^fl/fl^ mice than *Otud4*^fl/fl^ mice. The bacterial abundance in feces and cecum and the bacterial counts in spleen or liver were significantly reduced in *Def*-Cre;*Otud4*^fl/fl^ mice compared to *Otud4*^fl/fl^ mice (Fig. 4J). Expectedly, results from qRT-PCR analysis suggested that knockout of OTUD4 in Paneth cells promoted the expression of AMPs after *S*.t. infection (Fig. 4K). Collectively, these data demonstrate that OTUD4 in Paneth cells negatively regulates the expression of AMPs and promotes intestinal inflammation and bacterial infection.

### 2.6 OTUD4 inhibits the expression of AMPs in a MyD88-dependent manner

**Fig. 5:**
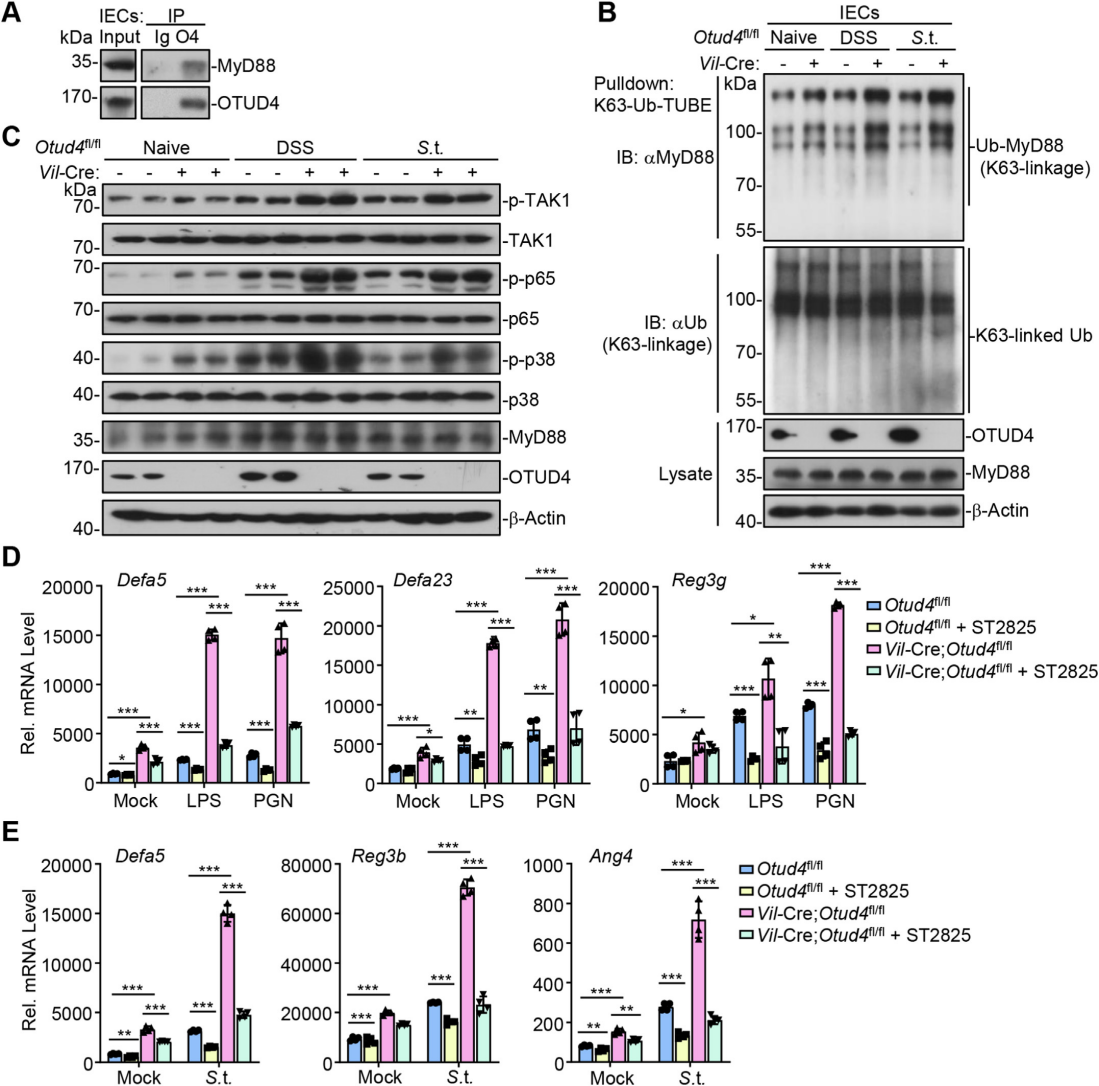
**OTUD4 inhibits the expression of AMPs by targeting MyD88.** (A) Whole-cell extract of IECs from wild-type C57BL/6 mice was immunoprecipitated with control IgG or anti-OTUD4 and subjected to immunoblot analysis. (B) Pulldown (with GST beads and GST-K63-Ub-TUBE) and immunoblot analysis of IECs from *Otud4*^fl/fl^ (n = 2) and *Vil*-Cre;*Otud4*^fl/fl^ (n = 2) mice that were uninduced, given 2.5% DSS in drinking water for 2 days or infected with *S*.t. (2 × 10^7^ c.f.u. per mouse) by gavage for 2 days. TUBE, tandem ubiquitin binding entity. (C) Immunoblot analysis of the indicated proteins in IECs from *Otud4*^fl/fl^ (n = 2) and *Vil*-Cre;*Otud4*^fl/fl^ (n = 2) mice treated as in (B). (D) qRT-PCR analysis of the indicated genes of intestinal organoids from *Otud4*^fl/fl^ and *Vil*-Cre;*Otud4*^fl/fl^ mice that were unstimulated or stimulated with LPS (10 μg/mL) or PGN (10 μg/mL) in the presence or absence of ST2825 (a MyD88 inhibitor, 10 μM) for 4 h. (E) qRT-PCR analysis of the indicated genes of intestinal organoids from *Otud4*^fl/fl^ and *Vil*-Cre;*Otud4*^fl/fl^ mice that were uninfected and infected with *S*.t. (MOI, 1:200) in the presence or absence of ST2825 (10 μM) for 2 h ∗*P* ​< ​0.05, ∗∗*P* ​< ​0.01, ∗∗∗*P* < 0.001 (Student's unpaired *t*-test in D, E). Graphs show mean ± S.D. (D, E). Data are representative of two independent experiments (A, C, D, E) or three independent experiments (B).

Both Wnt and MyD88 signaling pathways have been reported to regulate the expression of AMPs in the intestinal epithelium. However, we found that the Wnt signaling pathway or the expression of Wnt-related genes was not affected by knockout of OTUD4 in IECs after DSS treatment (Figs. S6A–C). In addition, the expression levels of AMPs, *Tcf7*, *Ctnnb1*, or *Gsk3b* were comparable between *Vil*-Cre;*Otud4*^fl/fl^ and *Otud4*^fl/fl^ intestinal organoids after Wnt3a stimulation (Fig. S6D), indicating that OTUD4 is not involved in Wnt signaling. It has been demonstrated that OTUD4 negatively regulates MyD88-mediated pathways by deconjugating K63-linked polyubiquitin chains from MyD88 (Zhao, et al., 2018), we hypothesized that OTUD4 might inhibit the expression of AMPs by targeting MyD88 in IECs. In co-immunoprecipitation assays, we found that OTUD4 constitutively interacted with MyD88 in IECs (Fig. 5A). In addition, K63-linked ubiquitination of MyD88 was substantially potentiated in the IECs of *Vil*-Cre;*Otud4*^fl/fl^ mice compared to *Otud4*^fl/fl^ mice after DSS treatment or *S*.t. infection (Fig. 5B), indicating that OTUD4 interacts with MyD88 and negatively regulates K63-linked ubiquitination of MyD88 in the intestine epithelium. Previous studies have shown that K63-linked ubiquitination of MyD88 promotes the activation of NF-κB and MAPKs that are involved in the production of AMPs (Chen et al., 2020; Muniz et al., 2012). Consistently, we found that knockout of OTUD4 in IECs increased the phosphorylation of TAK1, p65 and p38 after DSS treatment or *S*.t. infection (Fig. 5C), indicating that OTUD4 restricts the activation of NF-κB and MAPKs in IECs during gut inflammation or bacterial infection.

Lipopolysaccharide (LPS) and peptidoglycan (PGN) are essential components of the cell wall of bacteria that activate MyD88-dependent signaling through Toll-like receptors (Takeuchi & Akira, 2010). To examine whether OTUD4 regulated the expression of AMPs through MyD88, we prepared small intestinal organoids with the crypts from *Vil*-Cre;*Otud4*^fl/fl^ and *Otud4*^fl/fl^ mice and stimulated the organoids with LPS or PGN in the presence or absence of the MyD88 inhibitor (ST2825) followed by qRT-PCR assays. The results suggested that LPS- or PGN-induced expression of *Defa5*, *Defa23* and *Reg3g* was significantly higher in OTUD4-deficient organoids than in OTUD4-sufficient organoids and such upregulation was abrogated by ST2825 (Fig. 5D). Similarly, knockout of OTUD4 potentiated *S*.t. infection-induced expression of AMPs such as *Defa5*, *Reg3b* and *Ang4*, which was attenuated by ST2825 (Fig. 5E). These data indicate that OTUD4 restricts the expression of AMPs through MyD88 after bacterial infection.

It should be noted that the basal phosphorylation of TAK1, p65, and p38 was higher in *Vil*-Cre;*Otud4*^fl/fl^ IECs than in *Otud4*^fl/fl^ IECs (Fig. 5C). Interestingly, the basal K63-linked ubiquitination of MyD88 in IECs was also slightly increased by knockout of OTUD4 (Fig. 5B), which was consistent with the notion that OTUD4 constitutively interacted with MyD88 in IECs (Fig. 5A). In *in vitro* generated intestinal organoids, the basal expression of AMPs was also higher in *Vil*-Cre;*Otud4*^fl/fl^ organoids than in *Otud4*^fl/fl^ organoids, which was impaired by ST2825 (Fig. 5D and E and Fig. S6D), indicating that OTUD4 constitutively interacts with MyD88 and inhibits MyD88-dependent signaling and expression of AMPs. In this context, the results from 16S rRNA sequencing analysis suggested that more microbes of the phylum Firmicutes and fewer microbes of the phylum Proteobacteria existed in the feces of *Vil*-Cre;*Otud4*^fl/fl^ mice than *Otud4*^fl/fl^ mice under homeostatic conditions, although the diversity of bacterial flora was comparable (Fig. 2. G–I). Taken together, these results suggest that OTUD4 negatively regulates K63-linked ubiquitination of MyD88 and MyD88-dependent activation of NF-κB and MAPKs, thereby restricting the expression of AMPs in IECs and promoting bacterial infection and inflammation in the gut.

## 3 Discussion

Although the functions of OTUD4 in multiple biological processes including innate immune response have been investigated, it is unclear whether and how OTUD4 regulates intestinal inflammation and infection. Here in this study, we have demonstrated that OTUD4 inhibits the expression of AMPs in Paneth cells and supports intestinal inflammation and bacterial infection through deubiquitinating MyD88. By analyzing the transcriptomic data from the colonic biopsies of UC patients, we found that OTUD4 was highly expressed in the actively inflamed mucosa compared to remissive non-inflamed mucosal tissues or the normal mucosa from healthy individuals. In addition, the expression of OTUD4 was significantly increased in IECs from DSS-induced mice. Interestingly, we found that *Vil*-Cre- and *Def*-Cre- but not *Lyz2*-Cre-mediated deletion of OTUD4 in the *Otud4*^fl/fl^ mice alleviated DSS-induced colitis, indicating that OTUD4 in IECs or Paneth cells promotes intestinal inflammation. In addition, *Vil*-Cre;*Otud4*^fl/fl^ mice and *Def*-Cre;*Otud4*^fl/fl^ mice exhibit hyper-resistance to *S*.t. infection compared to *Otud4*^fl/fl^ mice. Interestingly, OTUD4 deficiency in IECs or Paneth cells led to the upregulation of AMPs which might be responsible for the resistance to DSS-induced colitis and *S.*t. infection of *Vil*-Cre;*Otud4*^fl/fl^ or *Def*-Cre;*Otud4*^fl/fl^ mice. Mechanistically, knockout of OTUD4 resulted in increased K63-linked ubiquitination of MyD88, which subsequently promotes the activation of NF-κB and MAPKs and the expression of AMPs. These findings collectively suggest that OTUD4 in Paneth cells promotes experimental colitis and *S*.t. infections in a manner of MyD88-dependent regulation of AMPs production (Fig. S7), indicating OTUD4 as a potential target for the treatment of IBD and bacterial infection.

It has been well-recognized that long-standing intestinal inflammation increases the incidence and risk of colorectal cancer (Shah & Itzkowitz, 2022). However, knockout of OTUD4 had minimal effects on colon cancer development in the AOM/DSS model or the AOM/VP model. There might be several reasons behind the phenomena. Firstly, different concentrations of DSS were used in these two models (2.5% DSS for the colitis model and 2.0% DSS for the colon cancer model, respectively). Higher concentrations of DSS cause more severe epithelial damage and bacterial infections in the intestine, which would induce more robust TLR-MyD88 signaling that reciprocally triggers regulatory machinery by OTUD4. In this context, previous reports have shown that knockout of IL-36R promotes DSS (2.5%)-induced colitis but alleviates DSS (1.5–2%)-induced colon fibrosis (Scheibe et al., 2017, 2019). Secondly, OTUD4 deficiency might lead to the activation of pro-tumoral pathways in epithelial cells, as it has been shown that OTUD4 is downregulated in some tumor cells and that overexpression of OTUD4 sensitizes tumor cells to death by modulating AKT, p53 or pyroptosis pathways (Di et al., 2022; Wu et al., 2019; Zhao et al., 2020). A third possibility is that epithelial OTUD4 regulates the production of AMPs during colitis or bacterial infections, while OTUD4 in other types of cells functions in colon cancer progression. It is not surprising, as IRAK-M, an adaptor protein downstream TLRs, protects DSS-induced colitis but sustains colorectal cancerogenesis in the epithelial cells, whereas slows down AOM/DSS-induced colon cancer in hematopoietic cells (Kesselring et al., 2016). A thorough understanding of the role of OTUD4 in different types of cells during colon cancer development requires comprehensive studies in the future.

We found that the expression of proinflammatory cytokines and chemokines was comparable between IECs from *Vil*-Cre;*Otud4*^fl/fl^ and *Otud4*^fl/fl^ mice after DSS treatment, whereas the expression of AMPs was significantly upregulated in the intestinal epithelium of *Vil*-Cre;*Otud4*^fl/fl^ mice compared to *Otud4*^fl/fl^ mice. Consistent with the notion that a prominent role of AMPs is killing bacteria, *Vil*-Cre;*Otud4*^fl/fl^ mice exhibited less altered microbiota in the gut after DSS treatment and were more resistant to *S*.t. infection than *Otud4*^fl/fl^ mice. These data have demonstrated an essential role of OTUD4 in inhibiting AMP expression to promote bacterial infection and inflammation in the gut. In addition to antibacterial activities, AMPs also modulate immune regulation, angiogenesis, and wound healing (Zhang et al., 2021). It is of great interest to examine whether OTUD4 is involved in those biological processes by modulating AMP production in the future.

AMPs are primarily produced in Paneth cells in the small intestine (Ho et al., 2013; Lueschow & McElroy, 2020; Muniz et al., 2012). We observed that there were more Paneth cells in each crypt of *Vil*-Cre;*Otud4*^fl/fl^ mice than *Otud4*^fl/fl^ mice and that the numbers of Paneth cells in each crypt of *Def*-Cre;*Otud4*^fl/fl^ and *Otud4*^fl/fl^ mice were comparable before or after DSS treatment, indicating that OTUD4 is involved in the differentiation but not the maintenance of Paneth cells. Although the mechanisms by which OTUD4 regulated Paneth cell differentiation were unknown, such regulation might contribute to the increased basal expression of AMPs in the *Vil*-Cre;*Otud4*^fl/fl^ IECs or organoids compared to the *Otud4*^fl/fl^ counterparts. Interestingly, the *Def*-Cre;*Otud4*^fl/fl^ mice exhibited similar anti-inflammatory phenotypes as the *Vil*-Cre;*Otud4*^fl/fl^ mice, including resistance to DSS-induced colitis and *S*.t. infection, increased expression of AMPs in the epithelium of small intestine and decreased inflammation in the gut compared to *Otud4*^fl/fl^ mice, indicating that OTUD4 in Paneth cells plays a primary role for AMP expression during DSS-induced colitis and bacterial infection.

Previous studies have shown that the expression of AMPs is regulated by the Wnt signaling pathway and the MyD88-dependent signaling pathway (Gong et al., 2010; Gregorieff et al., 2005; Menendez et al., 2013; Wang et al., 2021). Results from our transcriptomic data indicated that the expression of Wnt-related genes was comparable in OTUD4-sufficient and deficient IECs from the inflamed intestines, indicating that the hyperexpression of AMPs by OTUD4 deficiency was unlikely due to hyper Wnt signaling. MyD88 is required for the activation of NF-κB and MAPK pathways to induce AMPs production in the gut and OTUD4 negatively regulates the MyD88-dependent signaling by deconjugating K63-linked polyubiquitin chains from MyD88 (Chen et al., 2020; Muniz et al., 2012; Zhao, et al., 2018). Consistently, knockout of OTUD4 in IECs promoted the K63-linked polyubiquitination of MyD88 and the activation of downstream NF-κB and MAPKs in inflamed IECs, and the inhibitor ST2825 for MyD88 inhibited the expression of AMPs both in *Vil*-Cre;*Otud4*^fl/fl^ and in *Otud4*^fl/fl^ intestinal organoids that were treated with LPS or PGN, or infected with *S*.t., indicating that MyD88 is the main target of OTUD4 in IECs in regard of AMP expression after DSS treatment or bacterial infection.

We also noted that at steady conditions, the K63-linked polyubiquitination of MyD88, the phosphorylation of NF-κB and MAPKs, and the expression of AMPs also increased in OTUD4-deficient IECs or organoids compared to OTUD4-sufficient counterparts. OTUD4 likely regulated basal commensal microbiota-induced MyD88-dependent activation of NF-κB and MAPKs and expression of AMPs, which may reshape the microbiota in the gut. In support of this notion, results from the 16S rRNA sequencing assays suggest that *Vil*-Cre;*Otud4*^fl/fl^ mice shaped more microbes of the phylum Firmicutes and fewer microbes of the phylum Proteobacteria in the gut compared to *Otud4*^fl/fl^ mice before or after DSS treatment. In contrast, there were more microbes of the phylum Bacteroides before DSS treatment and fewer microbes of the phylum Bacteroides after DSS treatment in the gut of *Vil*-Cre;*Otud4*^fl/fl^ mice compared to *Otud4*^fl/fl^ mice. Members of phylum Bacteroides and Firmicutes are generally beneficial and promote nutrient metabolism of the host, while many common pathogens associated with human diseases belong to the phylum Proteobacteria such as *Escherichia*, *Shigella*, *Salmonella*, and *Yersinia* (Jandhyala et al., 2015; Rizzatti et al., 2017; Simpson & Campbell, 2015). In addition, the pathogenic members of phylum Bacteroides travel from the gut to other parts of the host body to induce inflammation when the intestinal epithelial barrier is damaged (Zafar & Saier, 2021). Therefore, the *Vil*-Cre;*Otud4*^fl/fl^ mice exhibit a healthier gut microbiota composition both in a steady state and the inflammatory state than the *Otud4*^fl/fl^ mice. Meanwhile, the richness and diversity of the microbiota in the *Vil*-Cre;*Otud4*^fl/fl^ mice were significantly less decreased than the *Otud4*^fl/fl^ mice after DSS treatment. Taken together, these results suggest that OTUD4 affects the compositions of gut microbiota probably through regulating MyD88-dependent expression of AMPs.

## 4 Methods

### 4.1 Mice

The *Otud4*^fl/fl^ and *Lyz2-*Cre;*Otud4*^fl/fl^ mice were previously described (Liuyu et al., 2019). The *Vil*-Cre (T000142) and wild-type C57BL/6 mice were purchased from GemPharmatech. The *Trp53* ^fl/fl^ (#008462) mice were purchased from Jackson Lab. The *D*ef-Cre (Defensinα6-Cre) mice were kindly provided by Dr. Shu Zhu (University of Science and Technology of China) (Wang et al., 2021). All mice were housed in the specific pathogen-free animal facility at Wuhan University with a 12-h dark/12-h light cycle and fed with standard food and water. All animal experiments were completed in accordance with protocols approved by the Institutional Animal Care and Use Committee of Wuhan University (approval number: MRI2021-LACA10). Mice tail DNA was amplified by PCR to identify the mice genotypes, and the genotyping primers were as follows: *Otud4*^fl/fl^ forward: 5′-GTCTCATTCTTGGCCTCGT-3′, *Otud4*^fl/fl^ reverse: 5′-ACATGCTGGCAAAACATTCATC-3’; *Lyz2-*Cre forward: 5′-CTTGGGCTGCCAGAATTTCTC-3′, *Lyz2-*Cre reverse: 5′-CCCAGAAATGCCAGATTACG-3’; *Vil*-Cre forward: 5′-GTGTTTGGTTTGGTTTCCTCTGCATAAGA-3′, *Vil*-Cre reverse: 5′- GCAGGCAAATTTTGGTGTACGGTCA-3’; *Trp53*^fl/fl^ forward: 5′- GGTTAAACCCAGCTTGACCA-3’; *Trp53*^fl/fl^ reverse: 5′- GGAGGCAGAGACAGTTGGAG-3’;*D*ef-Cre forward: 5′- AGGATAACAGCATCTCCCAGTTC-3′, *D*ef-Cre reverse:5′- ACTTCATCAGAGGTGGCATCC-3’.

### 4.2 DSS-induced colitis and colon cancer models

The experiments were performed as previously described (An et al., 2022; Wang et al., 2020; Yang et al., 2022). For the DSS-induced colitis model, eight-week-old mice were given 2.5% DSS (MP Biomedicals) in drinking water for 5 days followed by normal sterile water for 2 days before sacrifice. The Body weight, stool, and bleeding were daily observed and recorded. For the AOM/DSS colorectal tumor model, eight-week-old mice were injected i.p. with 10 mg per kg azoxymethane (AOM; Sigma), followed by treatment of 2.0% DSS drinking water for 7 days and regular water for 14 days. This cycle was repeated three times and mice were sacrificed 3 weeks after the end of the last DSS cycle. For the *Vil*-Cre;*Trp53*^fl/fl^ colon cancer model, 8-week-old mice were injected i.p. with AOM (10 mg per kg) once per week for 6 weeks successively. At the 20th week after the initial AOM injection, the mice were sacrificed for subsequent analysis. The mice of different genotypes were co-housed after weaning until the end of the experiments.

### 4.3 Hematoxylin and eosin (H&E) staining

The experiments were performed as previously described (An et al., 2022; Wang et al., 2020; Yang et al., 2022). Colon tissues paraffin blocks from mice were sectioned (6 μm) for H&E staining (Beyotime Biotech) followed by cover-slipping. Aperio VERSA 8 (Leica) multifunctional scanner was used to acquire images. Pathogenic scores of colitis were evaluated by combining disease activity index (DAI) score and histopathological score. DAI score was assessed for each animal as a cumulative score for severity of colitis, according to stool consistency (score: 0, normal; 1, soft and shaped; 2, loose stools; 3, diarrhea), rectal bleeding (score: 0, normal; 1 and 2, bloody stool; 3, gross bleeding) and body weight loss (score: 0, none; 1, 1%–5%; 2, 5%–10%; 3, 10%–15%; 4, >15%). Histopathological changes were analyzed as a cumulative score, on the basis of epithelial damage (0, normal morphology; 1, loss of goblet cells; 2, loss of goblet cells in large areas; 3, loss of crypts; 4, loss of crypts in large areas) and inflammatory cell infiltration (0, no infiltration; 1, infiltration around crypt bases; 2, infiltration spreading to muscularis mucosa; 3, extensive infiltration in the muscularis mucosa with abundant edema; 4, infiltration spreading to submucosa).

### 4.4 Isolation of intestinal epithelial cells

Small intestines were excised and flushed thoroughly with phosphate-buffered saline (PBS). They were turned inside out and cut into ∼0.5 cm sections. The sections were incubated in epithelial cell isolation buffer (RPMI 1640 medium including 2 mM EDTA, 1 mM DTT, 50 μg/mL gentamicin, 10% FBS) and shaken at 180 rpm at 37 °C for 30 min. The suspensions were filtered through a sterile nylon membrane and the flow-through was centrifuged at 1000 rpm at 4 °C for 5 min and saved as crude epithelial cells. The CD45^+^ positive selection kit (BioLegend) was used to remove immune cells and obtain purer epithelial cells.

### 4.5 qRT-PCR assay

Total RNA was extracted from cells using TRIzol (Invitrogen), and the first strand of cDNA was reverse transcribed using All-in-One cDNA Synthesis SuperMix (Biotool). After RT, qPCR (2 × SYBR Green Fast qRT-PCR Master Mix, Biotool) was performed using the CFX Real-Time System (Bio-Rad). The value obtained from each gene was normalized to that of the gene encoding β-actin. The sequences of primers used for the detection of mRNA transcripts were listed in Table S1.

### 4.6 Immunohistochemistry (IHC) assay

The experiments were performed as previously described (An et al., 2022; Wang et al., 2020; Yang et al., 2022). The paraffin sections of colon tissue underwent gradient dehydration and were treated with 1 mM EDTA (pH 8.0) in a microwave oven for 30 min for antigenic repair. The sections were incubated with primary antibodies overnight at 4 °C followed by a General purpose SP Kit (ZSGB-Bio) and DAB immunochromogenic reagent (Gene Tech) according to the manufacturer's instructions. The sections were then stained with hematoxylin (Beyotime Biotech). Signals were imaged with an Aperio VERSA 8 (Leica) multifunctional scanner. Primary antibodies used were anti-OTUD4 (Abcam, ab106368), anti-lysozyme (Abcam, ab108508), and anti-Ki67 (CST, 12202S).

### 4.7 Albian Blue PAS staining

The paraffin sections of colon tissue undergone gradient dehydration were stained with the Alcian Blue PAS Stain Kit (Abcam, ab245876). According to the manufacturer's instructions, Acetic Acid Solution (3%), Alcian Blue (pH 2.5) solution, Periodic Acid Solution, Schiff's Solution, and Hematoxylin were sequentially applied to tissue sections. Finally, the paraffin sections were dehydrated through graded alcohols and mounted in synthetic resin. The Aperio VERSA 8 (Leica) multifunctional scanner was used to acquire images.

### 4.8 mRNA transcriptome sequencing

Intestinal epithelial cells isolated from 2.5% DSS-induced mice were homogenized in 500 μL TRIzol (Invitrogen). Total RNAs were prepared and the qualities of RNAs were determined by agarose gel electrophoresis and spectrophotometer analysis. RNA-Sequencing (RNA-Seq) library preparation and sequencing for IEC samples were performed by Personalbio on the Illumina NovaSeq 6000 platform with Illumina HiSeq PE 150 strategy. mRNA-seq data were quality controlled by FastQc (v 0.11.8) and adapter bases placed in the 3’ end were trimmed by Cutadapt (v 1.1.5). RNA-Seq fastq files were aligned to the mouse genome (GRCm39) assembly by HISAT2 (v 2.0.5) with a maximum of two mismatches per read. Gene expression level was quantified by HT-seq (v 0.9.1) with default parameters and normalized by FPKM. Gene ontology analyses were performed using DAVID (https://david.ncifcrf.gov). The transcriptomic data and the gene set used for GSEA were included in Tables S2 and S3 (Supporting Information).

### 4.9 16S rRNA sequencing

Mice of different genotypes were housed separately and fed with 2.5% DSS for 5 d. The feces of mice were collected using sterilized cotton swabs into sterile centrifugal tubes before or after DSS treatment. Total genomic DNA samples were extracted from feces using the OMEGA Soil DNA Kit (Omega Bio-Tek). PCR amplification of the bacterial 16S rRNA genes V3–V4 region was performed using the forward primer 338F (5′-ACTCCTACGGGAGGCAGCA-3′) and the reverse primer 806R (5′-GGACTACHVGGGTWTCTAAT-3′). PCR amplicons were purified with Vazyme VAHTSTM DNA Clean Beads (Vazyme) and quantified using the Quant-iT PicoGreen dsDNA Assay Kit (Invitrogen). Sequencing libraries were generated using the Illlumina NovaSeq platform with NovaSeq 6000 SP Reagent Kit (500 cycles). Sequence data analyses were mainly performed using QIIME2 and R packages (v3.2.0).

### 4.10 *Salmonella* typhimurium infection and bacterial count analysis

*Salmonella* typhimurium (*S.*t.) was described previously (Li et al., 2013; Wang et al., 2020) and kindly provided by Dr. Shan Li (Huazhong Agricultural University). The mice of different genotypes were co-housed after weaning until the end of the experiments. Mice were injected by gavage with approximately 2 × 10^7^ c.f.u. of *S.*t. suspended in 200 μl of sterile PBS. The survival and weight of mice were monitored daily. At indicated time points after infection, the feces were collected with sterile cotton swabs, and the tissues (small intestine, colon, cecum, liver, and spleen) of the sacrificed mice were removed and weighed for further analysis. To calculate bacterial CFU from the fresh feces, cecum, liver, and spleen of infected mice, feces and tissues were homogenized with High throughput tissue lyser (Scientz-48) in PBS followed by centrifuge at 50 g for 1 min. The bacteria-containing supernatants were serially diluted and placed on conditional LB agar streptomycin plates. The bacterial colonies were counted after incubation at 37 °C for 24 h.

### 4.11 Immunoblot and co-immunoprecipitation assays

The experiments were performed as previously described (Xiong et al., 2022; Zhang et al., 2020a, 2020b). For the immunoblot assays, the Intestinal epithelial cells were homogenized with NP-40 lysis buffer (150 mM NaCl, 1 mM EDTA, 1% nonidet P-40) supplemented with proteinase and phosphatase inhibitors (Biotool). The lysates were cleared by centrifuge for 10 min at 4 °C. The supernatants were quantified and loaded to 10% sodium dodecyl sulfate–polyacrylamide gel (SDS-PAGE) for electrophoresis and transferred onto nitrocellulose membranes. Blocking was performed in 5% BSA in TBS for 1 h, and membranes were incubated with primary antibodies overnight at 4 °C. Membranes were incubated with horseradish peroxidase-conjugated secondary antibody for 1 h, and proteins were visualized by using ECL substrate. For the co-immunoprecipitation assays, the intestinal epithelial cell lysates were incubated with control IgG or anti-OTUD4 and protein G agarose overnight. The immunoprecipitates were washed three times with 1 ml prelysis buffer and subject to immunoblot analysis. Primary antibodies used were anti-OTUD4 (Bethyl Laboratories, A304-606A), anti-β-Actin (Abclonal, AC038), anti-MyD88 (Abclonal, A0980), anti-ubiquitin K63-specific linkage (Boston Biochem, UC318), anti-TAK1 (Abclonal, A19077), anti-Phosoho-TAK1 (Abclonal, A9437), anti-p65 (Santa Cruz Biotechnology, Sc372X), anti-Phosoho-p65 (CST, 3033S), anti-p38 (Santa Cruz Biotechnology, Sc7149), and anti-Phosoho-p38 (CST, 9216S).

### 4.12 TUBE pull-downs assay

GST-K63-Ub-TUBE (tandem ubiquitin binding entity) which consists of tandem ubiquitin binding domains and specifically binds poly K63-linked ubiquitin chains was expressed in *E. Coli* (DE3) and purified with a GST column as previously described (Ye et al., 2019). To analyze the ubiquitination of MyD88, GST-K63-Ub-TUBE and GST beads were incubated with the lysates of small intestinal epithelial cells at 4 °C overnight. The GST beads were washed twice with lysis buffer containing 500 mM NaCl and analyzed by SDS-PAGE, and then immunoblotted with the indicated antibodies.

### 4.13 Preparation of murine intestinal organoids

The small intestines of mice were rinsed with PBS and cut into 2–3 mm pieces. The pieces were vigorously shaken in pre-cooled PBS containing 2 mM EDTA for 1 h at 4 °C, and then EDTA was removed with cool PBS. Subsequently, the pieces were resuspended with 10 mL cool PBS and gently pipetted up and down three times. The supernatants containing the crypt suspension were filtered with 70 μm filters and transferred into a new 15 mL centrifuge tube. The precipitants were resuspended with cool PBS and pipetted up and down for repeated collection of crypts. Finally, four tubes of suspension were collected and observed under a microscope, the suspension with a large number of intact crypts was selected for centrifugation. Crypts were finally suspended in the Matrigel (Corning) and placed in the center of a well of a 24-well plate (40 μL per well). After the Matrigel had solidified (15 min at 37 °C), crypts were cultured in IntestiCult (Mouse) medium (Stem Cell) at 37 °C with 5% CO_2_. The culture medium was replaced every 2–3 d until experiments.

## Ethics statement

All mice were housed in the specific pathogen-free (SPF) facility. DSS induced colitis experiments and *Salmonella* typhimurium infection experiments were carried out in an SPF and ABSL-2 facility at the Medical Research Institute of Wuhan University, respectively. The experimental protocols followed the International Guiding Principles for Biomedical Involving Animals. The protocols for animal experiments were approved by the Institutional Animal Care and Use Committee of the Medical Research Institute of Wuhan University (approval number: MRI2021-LACA10).

## Statistical analysis

Differences between experimental and control groups were tested using Student's unpaired *t*-test. *P* values less than 0.05 were considered statistically significant. Kaplan-Meier method was used to analyze the survival of mice. Prism 8.3.0 was used to generate graphs and perform statistical analysis.

## Data availability

The transcriptome datasets generated during this study are available at Gene Expression Omnibus (GEO) under the superseries number GSE222836. The 16S sequencing datasets generated during this study are available at NCBI BioProject under the number: PRJNA927136. All other data supporting the findings of this study are available from the corresponding author upon reasonable request.

## Author contributions

B.Z. and D.L. supervised the study. K.Y. performed the major experiments. Y.-Y.G. helped with mouse breeding and genotyping. T.L. and P.W. helped with DSS-induced colitis modeling. B.Z., D.L., Z.-D.Z., and K.Y. wrote the paper. All the authors analyzed data.

## Conflict of interest

The authors declare no conflict of interest.
