# Supplementary material for: The deubiquitinase OTUD4 inhibits the expression of antimicrobial peptides in Paneth cells to support intestinal inflammation and bacterial infection
Source: Cell Insight. 2023 Apr 5;2(3):100100. doi: 10.1016/j.cellin.2023.100100 (PMC10123543; doi:10.1016/j.cellin.2023.100100)
Supplement: Multimedia component 1 [file mmc1.pdf]

Supporting Information

The deubiquitinase OTUD4 inhibits the expression of antimicrobial peptides in Paneth cells to support intestinal inflammation and bacterial infection

Yu et al

Figure S1

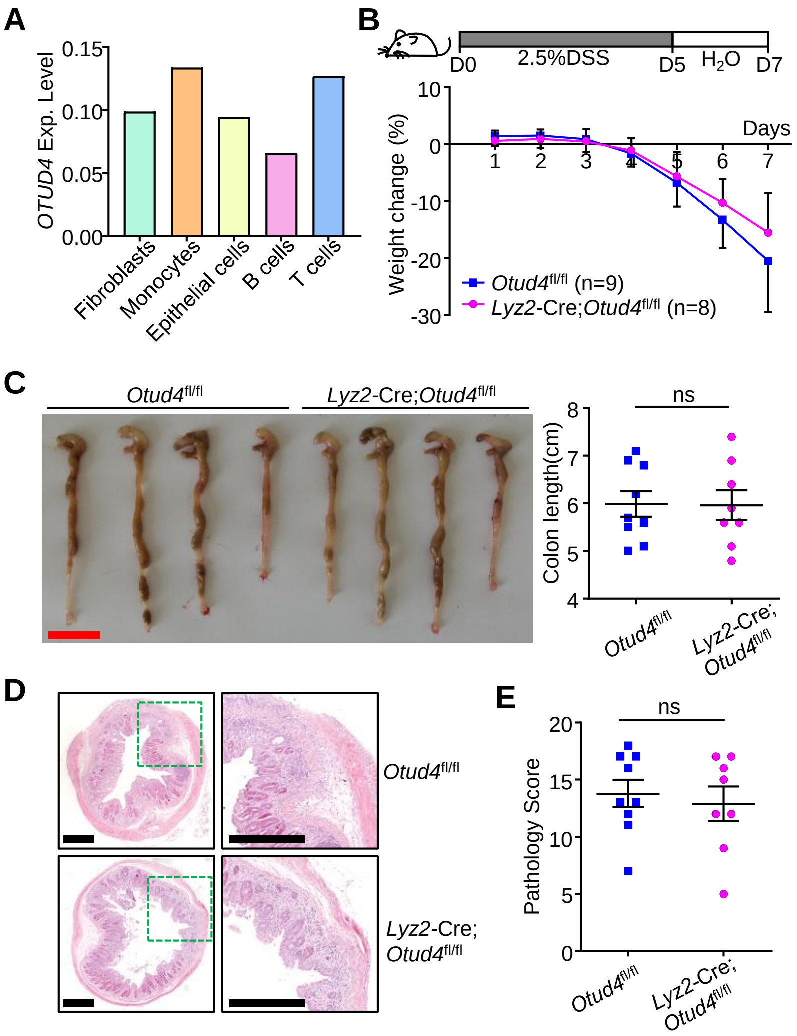

Figure S1 OTUD4 in myeloid cells is dispensable DSS-induced colitis.

(A) *OTUD4* mRNA expression levels in different types of colonic cells from the ulcerative mucosa of ulcerative colitis (UC) patients (https://portals.broadinstitute.org/single_cell, Single Cell Portal: SCP259).

(B) A scheme of DSS-induced colitis (upper scheme) and body weight change (lower graph) of *Otud4*$^{fl/fl}$ (n=9) and *Lyz2*-Cre;*Otud4*$^{fl/fl}$ (n=8) mice with 2.5% DSS for 5 days followed by normal sterile water for 2 days.

(C) A representative image and the lengths of colons of *Otud4*$^{fl/fl}$ (n=9) and *Lyz2*-Cre;*Otud4*$^{fl/fl}$ (n=8) mice treated as in (B).

(D) Representative images of HE-stained colon sections of *Otud4*$^{fl/fl}$ and *Lyz2*-Cre;*Otud4*$^{fl/fl}$ mice treated as in (B).

(E) The pathology scores of *Otud4*$^{fl/fl}$ (n=9) and *Lyz2*-Cre;*Otud4*$^{fl/fl}$ (n=8) mice treated as in (B).

$*P < 0.05$, $**P < 0.01$, $***P < 0.001$ (Student's unpaired *t*-test in B, C, E). Graphs show mean ± S.D. (B, C, E). Red and black scale bars represent 2cm and 400 µm (C, D). Data are combined two independent experiments (B, C, E) or representative of two independent experiments (D).

Figure S2

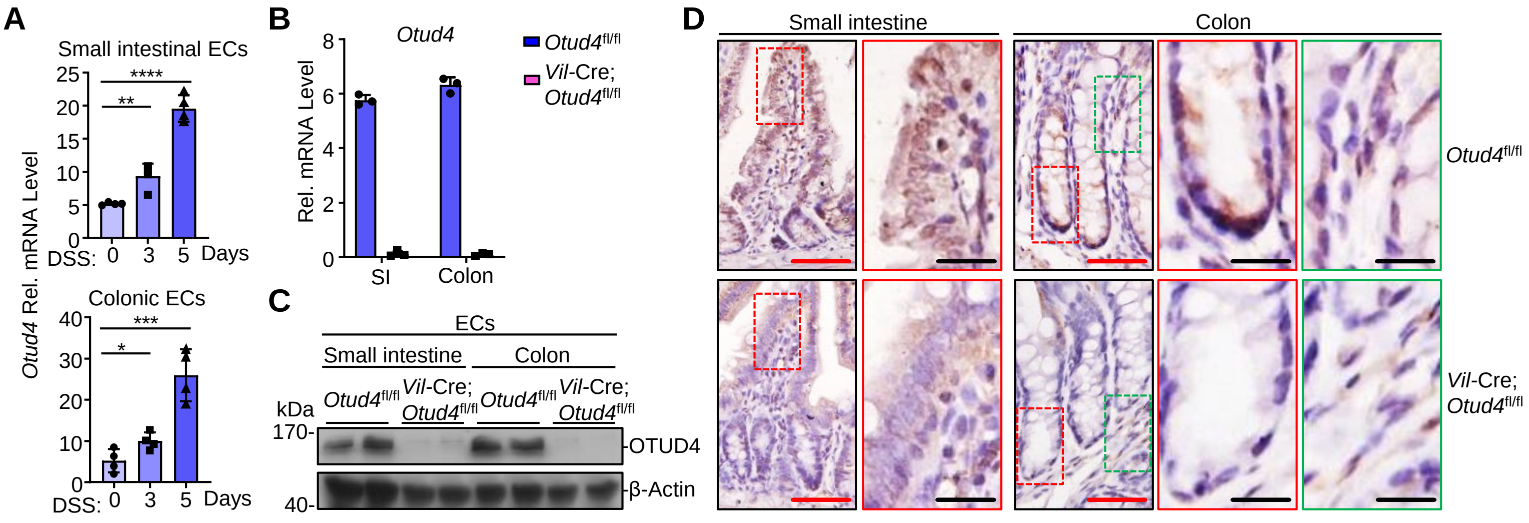

Figure S2 Analysis of OTUD4 IECs conditional knockout mice.

(A) qRT-PCR analysis of *Otud4* mRNA levels in epithelial cells (ECs) from small intestines (upper graph) and colons (lower graph) of *Otud4*$^{fl/fl}$ (n=4) mice treated with 2.5% DSS for 0, 3, 5 days.

(B) qRT-PCR analysis of *Otud4* mRNA levels in ECs from small intestines and colons of *Otud4*$^{fl/fl}$ (n=3) and *Vil*-Cre;*Otud4*$^{fl/fl}$ (n=3) mice.

(C) Immunoblot analysis of OTUD4 in ECs from small intestines and colons of *Otud4*$^{fl/fl}$ (n=2) and *Vil*-Cre;*Otud4*$^{fl/fl}$ (n=2) mice.

(D) IHC analysis of OTUD4 in small intestines and colons of *Otud4*$^{fl/fl}$ and *Vil*-Cre;*Otud4*$^{fl/fl}$ mice. Red and green rectangles indicate crypts and lamina propria, respectively.

*P < 0.05, **P < 0.01, ***P < 0.001 (Student's unpaired *t*-test in A). Graphs show mean ± S.D. (A, B). Red and black scale bars represent 60 μm and 20 μm, respectively. Data are representative of two independent experiments (A–D).

Figure S3

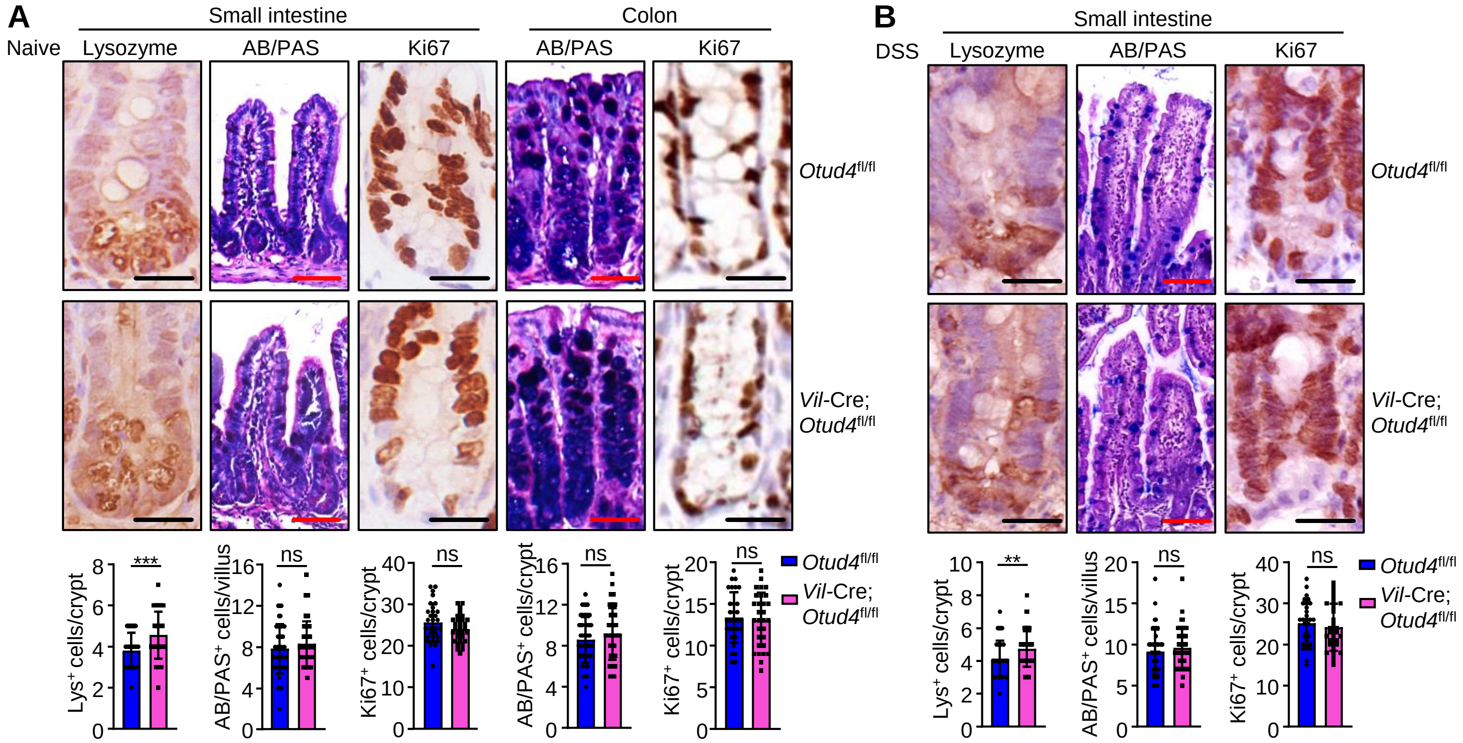

Figure S3 OTUD4 deficiency in IECs leads to an increase in Paneth cells.

(A) IHC analysis (upper images) and counts (lower graphs) of lysozyme (Lys$^+$) (n = 50 crypts), AB/PAS (n = 50 villi/crypts) or Ki67 (n = 30 crypts) staining of crypts or villi of *Otud4*$^{fl/fl}$ and *Vil*-Cre;*Otud4*$^{fl/fl}$ mice that were uninduced.

(B) IHC analysis (upper images) and counts (lower graphs) of lysozyme (Lys$^+$) (n = 50 crypts), AB/PAS (n = 50 villi) or Ki67 (n = 30 crypts) staining of crypts or villi of *Otud4*$^{fl/fl}$ and *Vil*-Cre;*Otud4*$^{fl/fl}$ mice that were treated with 2.5% DSS for 5 days followed by normal sterile water for 2 days.

**P* < 0.05, ***P* < 0.01, ****P* < 0.001 (Student's unpaired *t*-test in A, B). Graphs show mean ± S.D.(A, B). Red and black scale bars represent 100 μm and 20 μm, respectively.

Figure S4

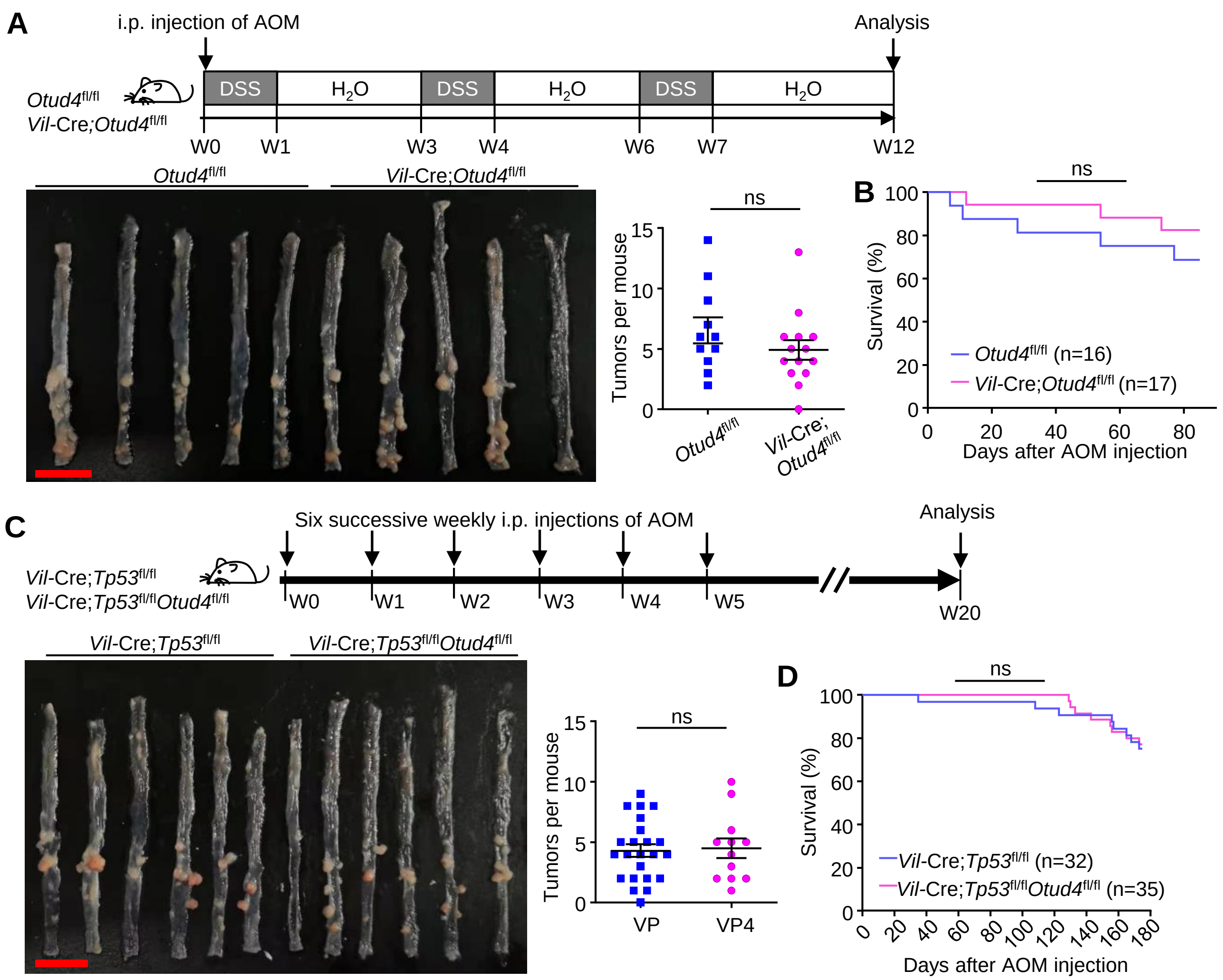

Figure S4 OTUD4 in IECs does not affect tumorigenesis in the colon.

(A) A scheme of AOM/DSS colon cancer model (upper scheme), a representative image (lower image) and tumor counts (lower graph) in colons of *Otud4*$^{fl/fl}$ (n=11) and *Vil*-Cre;*Otud4*$^{fl/fl}$ (n=14) mice that were induced with the AOM/DSS protocol.

(B) Survival of *Otud4*$^{fl/fl}$ (n=16) and *Vil*-Cre;*Otud4*$^{fl/fl}$ (n=17) mice that were induced with AOM/DSS colon cancer as in (A).

(C) A scheme of induction of colon tumors with the AOM/*Vil*-Cre;*Trp53*$^{fl/fl}$ model (upper scheme), representative image (lower image) and tumor counts (lower graph) in colons of *Vil*-Cre; *Trp53*$^{fl/fl}$ (n = 23) and *Vil*-Cre;*Trp53*$^{fl/fl}$*Otud4*$^{fl/fl}$ (n = 12) mice that were injected weekly with AOM (10 mg per kg body weight, i.p.) for 6 weeks successively and killed at the 20th week after initial AOM injection.

(D) Survival of *Vil*-Cre;*Trp53*$^{fl/fl}$ (n=32) and *Vil*-Cre;*Trp53*$^{fl/fl}$*Otud4*$^{fl/fl}$ (n=35) mice that were induced with colon cancer as in (C).

**P* < 0.05, ***P* < 0.01, ****P* < 0.001 (Log-rank test in B, D, Student's unpaired *t*-test in A, C). Graphs show mean ± S.D. (A, C). Scale bars represent 2cm (A, C) Data are combined two independent experiments (A–C) or four independent experiments (D).

Figure S5

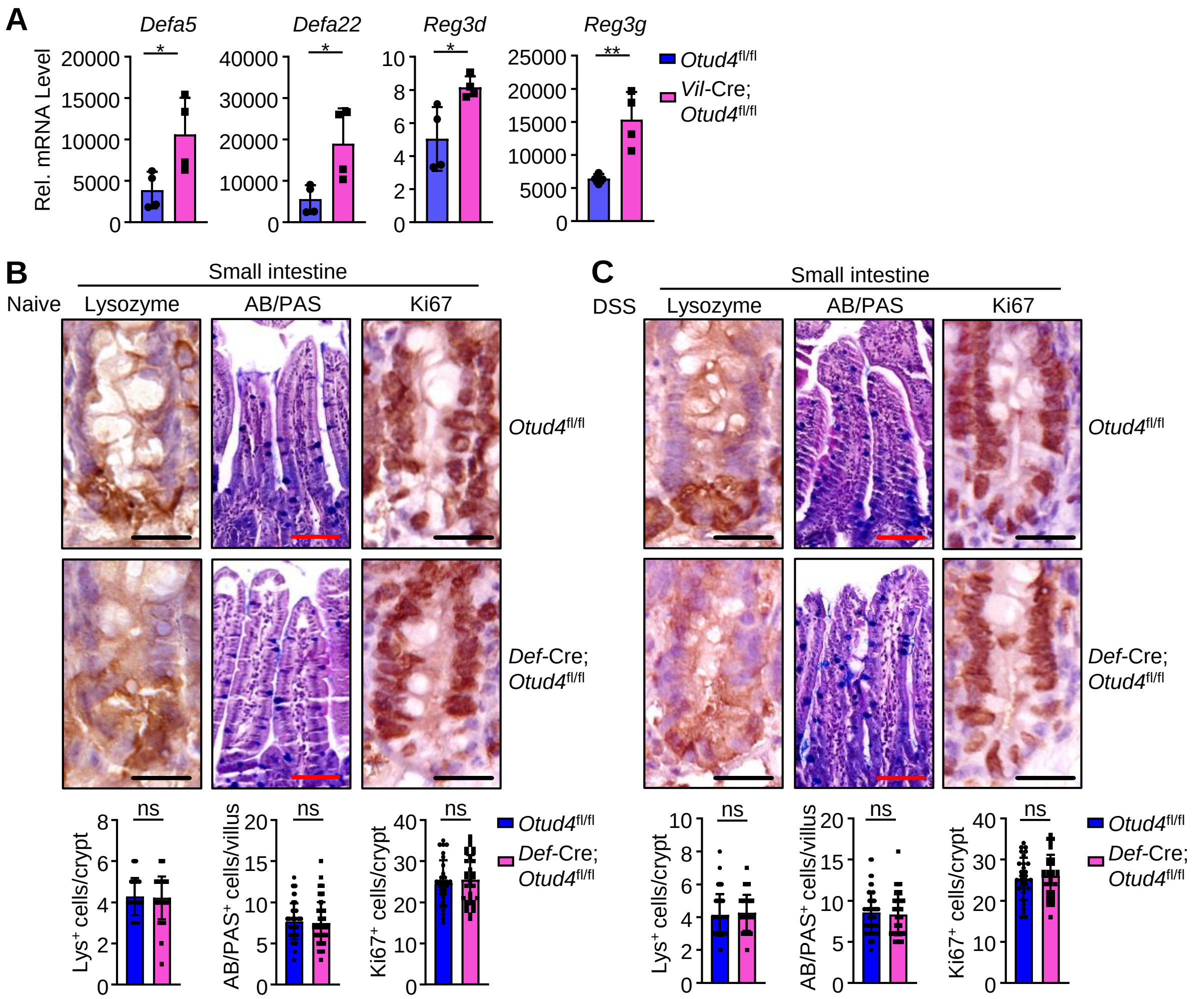

Figure S5 OTUD4 deficiency in Paneth cells does not alter the number of Paneth cell in crypts.

(A) qRT-PCR analysis of the indicated genes of IECs from two groups of *Otud4*$^{fl/fl}$ (n=4) and *Vil*-Cre;*Otud4*$^{fl/fl}$ (n=4) mice that were uninduced.

(B) IHC analysis (upper images) and counts (lower graphs) of lysozyme (Lys^{+}) (n = 50 crypts), AB/PAS (n = 50 villi) or Ki67 (n = 30 crypts) staining of crypts or villi of *Otud4*$^{fl/fl}$ and *Def*-Cre;*Otud4*$^{fl/fl}$ mice that were uninduced.

(C) IHC analysis (upper images) and counts (lower graphs) of lysozyme (Lys^{+}) (n = 50 crypts), AB/PAS (n = 50 villi) or Ki67 (n = 30 crypts) staining of crypts or villi of *Otud4*$^{fl/fl}$ and *Def*-Cre;*Otud4*$^{fl/fl}$ mice that were treated with 2.5% DSS for 5 days followed by normal sterile water for 2 days.

$^{*}P < 0.05$, $^{**}P < 0.01$, $^{***}P < 0.001$ (Student's unpaired *t*-test in A–C). Graphs show mean ± S.D.(A–C). Red and black scale bars represent 100 μm and 20 μm, respectively.

Figure S6

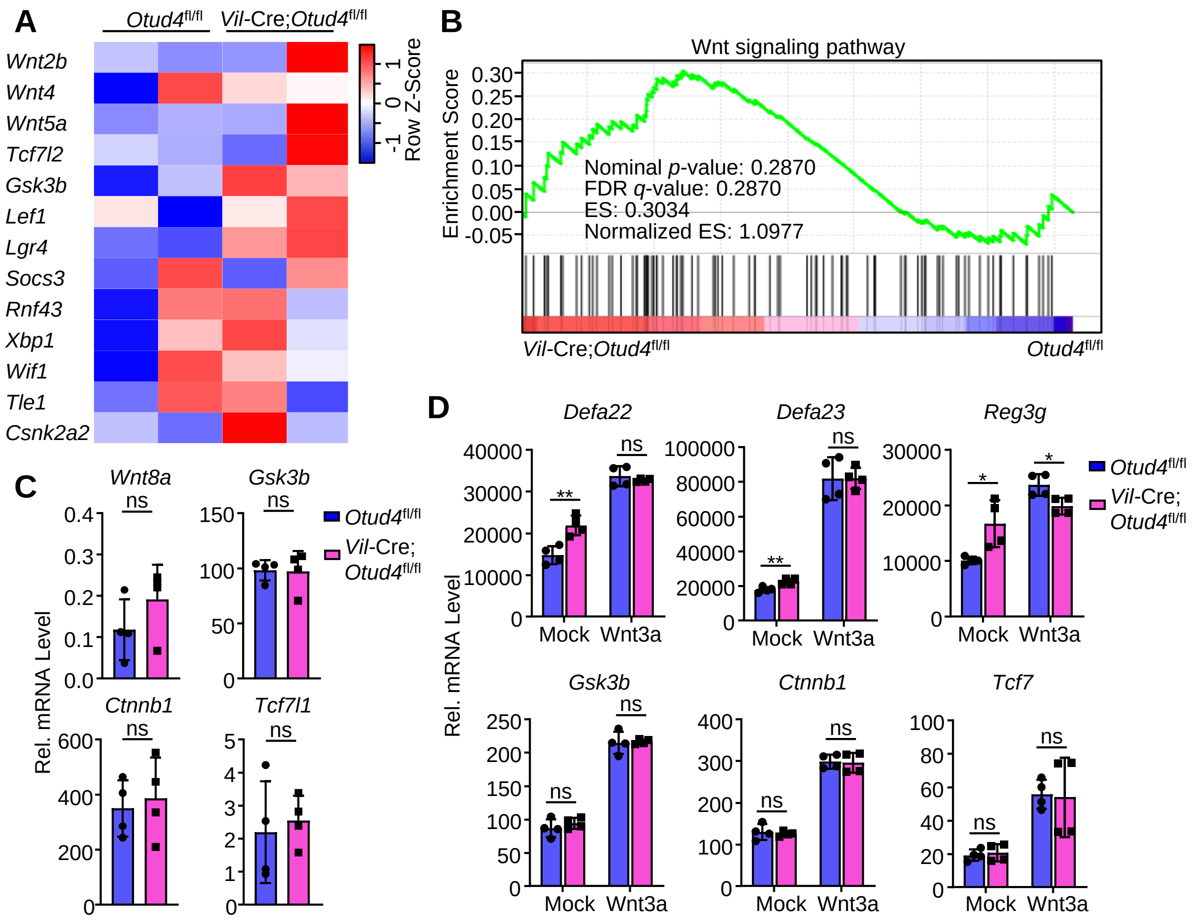

Figure S6 Regulation of AMPs production controlled by OTUD4 is Wnt-independent.

(A) Heatmap of the indicated genes related to Wnt signaling pathway from the transcriptomic profile of IECs from *Otud4*$^{fl/fl}$ (n=2) and *Vil*-Cre;*Otud4*$^{fl/fl}$ (n=2) mice that were given 2.5% DSS in drinking water for 2 d.

(B) GSEA analysis of Wnt signaling pathway from the transcriptomic data obtained in (A). FDR, false discovery rate; ES, enrichment score.

(C) qRT-PCR analysis of the indicated genes of IECs from two groups of *Otud4*$^{fl/fl}$ (n=4) and *Vil*-Cre;*Otud4*$^{fl/fl}$ (n=4) mice that were given 2.5% DSS in drinking water for 2 d.

(D) qRT-PCR analysis of the indicated genes of intestinal organoids from *Otud4*$^{fl/fl}$ and *Vil*-Cre;*Otud4*$^{fl/fl}$ mice that were unstimulated or stimulated with Wnt3a (100 ng/mL) for 4 h.

*P < 0.05, **P < 0.01, ***P < 0.001 (Student's unpaired *t*-test in C, D). Graphs show mean ± S.D. (C, D).

Figure S7

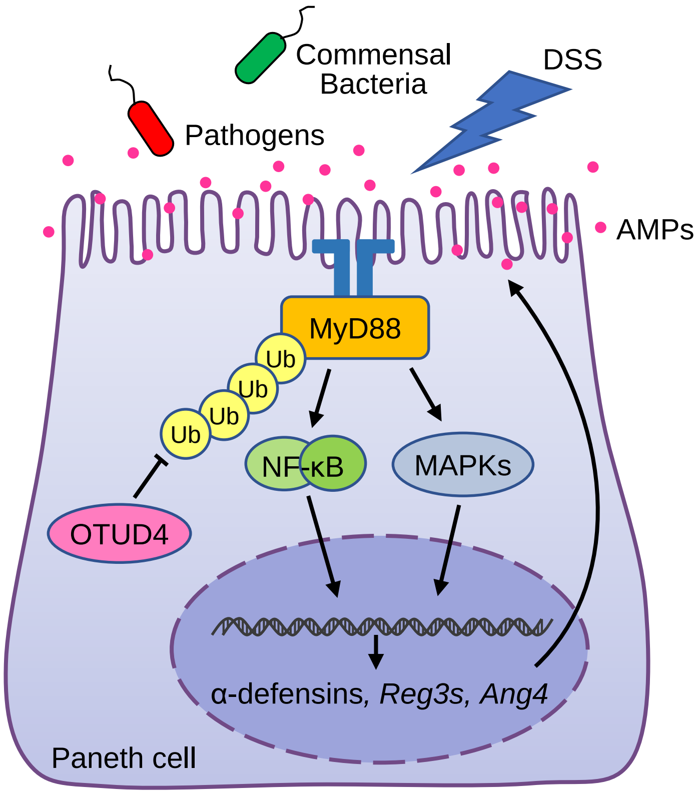

Figure S7 A model of OTUD4-mediated regulation of AMP production and intestinal inflammation in Paneth cells.
OTUD4 restricts the K63-linked polyubiquitination of MyD88 in Paneth cells, the activation of NF-κB and MAPKs and the expression of AMPs at homeostatic and inflammatory conditions, and thereby promotes gut inflammation caused by epithelial damage or bacterial infection.
