# Supplementary material for: The deubiquitinase OTUD4 inhibits the expression of antimicrobial peptides in Paneth cells to support intestinal inflammation and bacterial infection
Source: Cell Insight. 2023 Apr 5;2(3):100100. doi: 10.1016/j.cellin.2023.100100 (PMC10123543; doi:10.1016/j.cellin.2023.100100)
Supplement: Multimedia component 2 [file mmc2.docx]

Table S1. qPCR primer sequences

| Primers | Primer sequence (5’-3’) | | Primer sequence (3’-5’) | |
| --- | --- | --- | --- | --- |
| m*Actinb* | | ACGGCCAGGTCATCACTATT | | TGGCATAGAGGTCTTTACGGA |
| m*Otud4* | | GGAGGTTAGGATGGCCTGTATC | | GGCACTTATTTCCACTTGTCCT |
| m*Defa5* | | TTGGGCTCCTGCTCAACAAT | | GACACAGCCTGGTCCTCTTC |
| m*Defa20* | | GTCTCCTTTGGAGACCCAGAAG | | GCAGCAGAACAAAAGTCGTCCTG |
| m*Defa21* | | CCAGGGGAAGATGACCAGGCTG | | TGCAGCGACGATTTCTACAAAGGC |
| m*Defa22* | | ACGTCGCTGCAATAGAGGAG | | GCCTCAGAGCTGATGGTTGT |
| m*Defa23* | | AACTGAGGAGCAGCCAGGGAAA | | CCTCTTGTTCTACAATAGCATACC |
| m*Ang4* | | GGCACCAAGAAAAACATCAGGGC | | GTGCGTACAAGTGGTGATCTGG |
| m*Reg3d* | | GATTGGACTCCATGATCTGTCAC | | CAGTAACCGTGGTGTGCAGACA |
| m*Reg3g* | | CGTGCCTATGGCTCCTATTGCT | | TTCAGCGCCACTGAGCACAGAC |
| m*Reg3b* | | TGGCTCCTACTGCTATGCCTTG | | CGCTATTGAGCACAGATACGAGG |
| m*Wnt8a* | | GGTGACTTGGAAAACTGCGGCT | | CCAAACTGTCCACGAAGAGTCTG |
| m*Gsk3b* | | GAGCCACTGATTACACGTCCAG | | GAGCCACTGATTACACGTCCAG |
| m*Ctnnb1* | | GTTCGCCTTCATTATGGACTGCC | | ATAGCACCCTGTTCCCGCAAAG |
| m*Tcf7l1* | | CCTCTCATCACCTACAGCAACG | | CTGGAGACAGTGGGTAATACGG |
| m*Tcf7* | | CCTGCGGATATAGACAGCACTTC | | TGTCCAGGTACACCAGATCCCA |
